# Design and validation of recombinant protein standards for quantitative Western blot analysis of cannabinoid CB_1_ receptor density in cell membranes: an alternative to radioligand binding methods

**DOI:** 10.1186/s12934-022-01914-1

**Published:** 2022-09-15

**Authors:** Miquel Saumell-Esnaola, Ainhoa Elejaga-Jimeno, Leyre Echeazarra, Leire Borrega-Román, Sergio Barrondo, Maider López de Jesús, Imanol González-Burguera, Alberto Gómez-Caballero, María Aranzazu Goicolea, Joan Sallés, Gontzal García del Caño

**Affiliations:** 1 Department of Pharmacology, Faculty of Pharmacy, University of the Basque Country UPV/EHU, 01006 Vitoria-Gasteiz, Spain; 2 Bioaraba, Neurofarmacología Celular y Molecular, 01008 Vitoria-Gasteiz, Spain; 3 Department of Analytical Chemistry, Faculty of Pharmacy, University of the Basque Country UPV/EHU, 01006 Vitoria-Gasteiz, Spain; 4 Department of Physiology, Faculty of Pharmacy, University of the Basque Country UPV/EHU, 01006 Vitoria-Gasteiz, Spain; 5 Bioaraba, Dispositivos Móviles Para El Control de Enfermedades Crónicas, 01008 Vitoria-Gasteiz, Spain; 6 Centro de Investigación Biomédica en Red de Salud Mental (CIBERSAM), 28029 Madrid, Spain; 7 Department of Neurosciences, Faculty of Pharmacy, University of the Basque Country UPV/EHU, 01006 Vitoria-Gasteiz, Spain

**Keywords:** GPCR expression analysis, Quantitative Western blot, Radioligand saturation binding, Cannabinoid CB_1_ receptor antibodies, Carboxy-terminal tail, Soluble recombinant protein standards, GST fusion proteins

## Abstract

**Background:**

Replacement of radioligand binding assays with antibody-antigen interaction-based approaches for quantitative analysis of G protein-coupled receptor (GPCR) levels requires the use of purified protein standards containing the antigen. GPCRs in general and cannabinoid CB_1_ receptor in particular show a progressive tendency to aggregate and precipitate in aqueous solution outside of their biological context due to the low solubility that the hydrophobic nature imprinted by their seven transmembrane domains. This renders full-length recombinant GPCRs useless for analytical purposes, a problem that can be overcome by engineering soluble recombinant fragments of the receptor containing the antigen.

**Results:**

Here we generated highly soluble and stable recombinant protein constructs GST-CB1_414–472_ and GST-CB1_414-442_ containing much of the human CB_1_ receptor C-terminal tail for use as standard and negative control, respectively, in quantitative Western blot analysis of CB_1_ receptor expression on crude synaptosomes of the adult rat brain cortex. To this end we used three different antibodies, all raised against a peptide comprising the C-terminal residues 443–473 of the mouse CB_1_ receptor that corresponds to residues 442–472 in the human homolog. Estimated values of CB_1_ receptor density obtained by quantitative Western blot were of the same order of magnitude but slightly higher than values obtained by the radioligand saturation binding assay.

**Conclusions:**

Collectively, here we provide a suitable Western blot-based design as a simple, cost-effective and radioactivity-free alternative for the quantitative analysis of CB_1_ receptor expression, and potentially of any GPCR, in a variety of biological samples. The discrepancies between the results obtained by quantitative Western blot and radioligand saturation binding techniques are discussed in the context of their particular theoretical bases and methodological constraints.

**Supplementary Information:**

The online version contains supplementary material available at 10.1186/s12934-022-01914-1.

## Background

The radioligand binding assays have greatly fuelled the biochemical identification and the pharmacological characterization of members of the G protein-coupled receptor (GPCR) superfamily as drug targets. Thus, this technique has been the gold standard to characterize in vitro ligand-receptor interactions and also to quantify receptor density in plasma membrane and/or different subcellular compartments [1–3]. Radioligand binding assays are applicable to any receptor of interest, provided a selective radioactively labeled ligand is available. However, the relative ease/simplicity of the assay sometimes leads to its misuse because factors that can affect radioligand binding parameters (specific radioactivity, type and ionic strength of the buffer, presence of mono and divalent ions, or temperature) are ignored [1–4]. Furthermore, estimation of maximum binding sites (*B*_*max*_) parameter used to define the GPCR density by radioligand saturation binding assays depends on the efficacy of the ligand used as agonist, inverse agonist or neutral antagonist [5, 6]. In fact, according to the GPCR signaling conformational model [7, 8], only neutral antagonists show high affinity for any conformational state of GPCRs, whereas agonists and inverse agonists can selectively bind and stabilize G protein-coupled and uncoupled receptors, respectively, which can skew the estimate of receptor density [5]. In addition to these technical challenges, other drawbacks of using radioligands include health risks, the need for well-controlled designated areas and highly qualified technical personnel and the generation of radioactive toxic waste. Over time, these limitations and caveats have led pharmaceutical companies working in the field of GPCR drug discovery to move to designs that avoid the use of radioactive elements and allow high-throughput automated characterization of drug-receptor interaction, such as fluorescence polarization, fluorescence resonance energy transfer or surface plasmon resonance [9]. The sharp decline in demand for radioligands by the pharmaceutical industry and the progressively decreasing number of suppliers and distributors has led to a drastic increase in radioligand prices, ultimately making radioligand binding techniques economically prohibitive in many academic/university laboratories.

Among GPCRs, the cannabinoid receptor type 1 (CB_1_ receptor) is the most abundant one in the mammalian brain [10–14] and has been extensively studied during the last decades using radioligand binding techniques. The contribution of these studies has been essential to formulate etiopathogenic hypotheses and to propose the CB_1_ receptor as a promising therapeutic target [15, 16] for highly prevalent neuropathological conditions. For example, the radioligand-based studies have revealed a dysregulation of the CB_1_ receptor in a variety of neurological diseases such as schizophrenia [17–19], depression [20], drug abuse [21–23], epilepsy [24] or malignant astrocytoma [25], both in animal models and human. Despite its wide use in basic and preclinical research, methodological and cost limitations of the radioligand binding approaches mentioned above have led many researchers to replace it with Western blot, which is generally used as a semi-quantitative method to compare protein expression between different conditions or samples. Nonetheless, Western blot can be adapted for quantitative determination of GPCR density in a variety of biological samples, provided there are both highly selective antibodies and standard proteins containing the amino acid sequence used as immunogen [26–29], as is the case for the CB_1_ receptor [30, 31]. However, there are no studies that address the quantitative determination of the CB_1_ receptor expression by Western blot. A probable cause for this may lie in the difficulty of expressing recombinant GPCRs in bacterial systems, along with the tendency of these receptors to aggregate due to their hydrophobic nature [32], which constitutes a true obstacle to the storage in aqueous media. As Martin and colleagues have elegantly demonstrated [29], this problem can be solved by using standards consisting of polypeptide fragments of the target protein containing the immunogen. The CB_1_ receptor is a seven-transmembrane receptor [33–35] whose carboxy-terminal (C-terminal) tail facing the aqueous intracellular side possesses two unique properties that make it suitable to be used as a protein standard for quantitative Western blotting. On the one hand, it is predictable that it is stable in aqueous solution and, on the other hand, peptide sequences at its extreme end are against which the most reliable antibodies for the detection of the CB_1_ receptor have been generated [14, 30, 36–38]. Here, we designed, produced and purified recombinant protein standards comprising fragments of the cytosolic tail of the human CB_1_ receptor and proved their suitability for the analysis of receptor density in cell membranes of the rat cerebral cortex by quantitative immunoblotting, as an alternative procedure to the use of radioligand binding assays.

## Results

### Generation of constructs encoding GST-fusion proteins

**Fig. 1:**
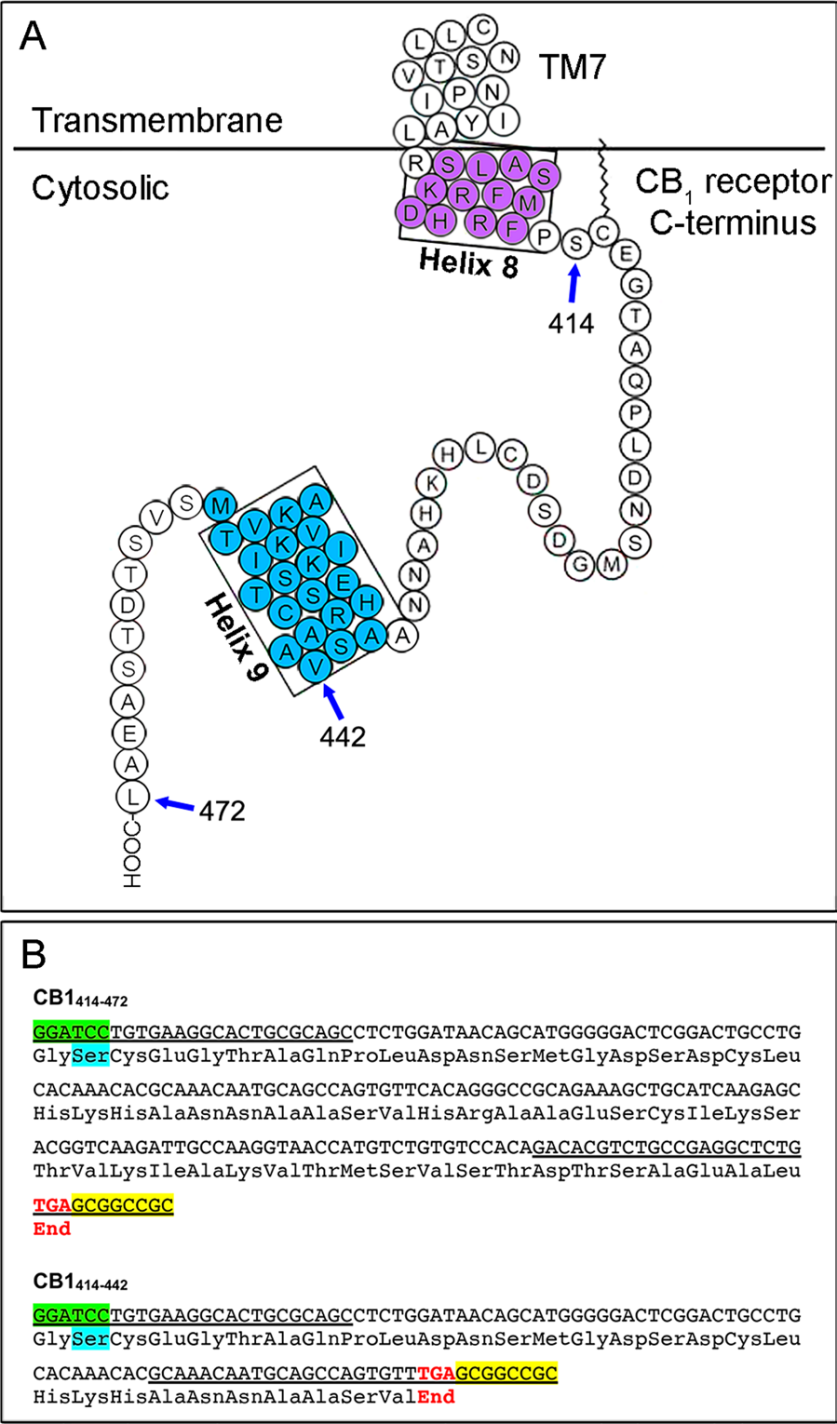
Design for the engineering of recombinant DNA for subsequent insertion into the pGEX-6P1 plasmid. **A** Schematic representation of the cytosolic tail of the human CB_1_ receptor showing its primary amino acid sequence. The helix 8 and helix 9 structural domains are highlighted in violet and cyan, respectively. The arrows indicate the initial and terminal amino acids of the polypeptides ranging from serine 414 to terminal lysine 472 (CB1_414–472_) and from serine 414 to valine 442 (CB1_414–442_) chosen for the design of the fusion proteins GST-CB1_414–472_ and GST-CB1_414–442_. Illustration modified from Stadel and Kendall [64]. **B** DNA products obtained by PCR amplification and the encoded polypeptides CB1_414–472_ and CB1_414–442_. The underlined DNA sequences indicate the hybridization sites for the Fw and Rv primers. The BamHI and NotI restriction sites for cloning into the pGEX-6P1 vector are highlighted in green and yellow, respectively. The triplets encoding serine 414 in the human CB1 receptor and the stop codons are highlighted in cyan and red, respectively

**Fig. 2:**
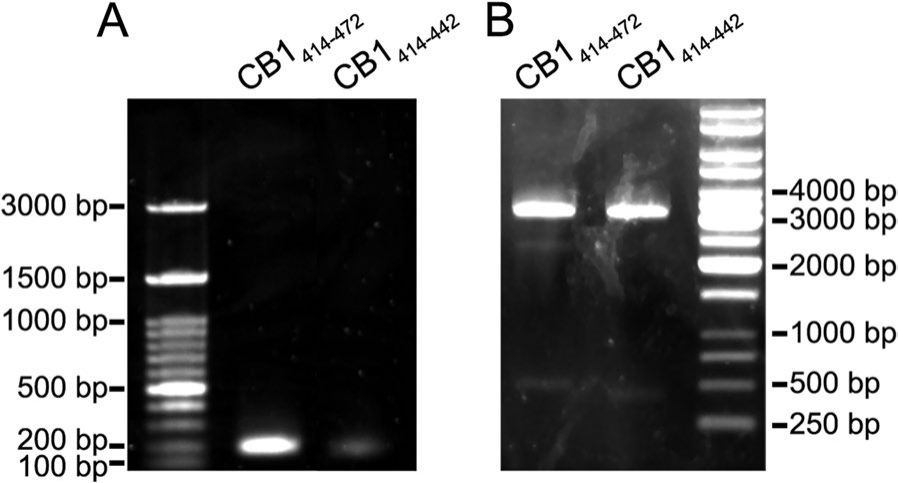
Agarose gel electrophoresis of PCR products and transformants.** A** Agarose gel electrophoresis analysis of PCR amplicons coding for CB1_414-472_ and CB1_414–442_ fragments of the cytosolic tail of the human CB_1_ receptor with expected sizes of 191 and 101 bp, respectively. **B** BamHI/BsrGI restriction analysis of bacterial clones transformed with pCR-Blunt II-TOPO^™^ vector containing DNA inserts coding for CB1_414–472_ and CB1_414–442_ polypeptides. The restriction maps are consistent with DNA fragments inserted in forward orientation. Expected sizes (bp) of restriction fragments were 3174/493/43 and 3174/403/43 for plasmid with DNA inserts coding for CB1_414–472_ and CB1_414–442_, respectively

PCR amplification of DNA sequences coding for CB1_414–472_ and CB1_414–442_ fragments of the cytosolic tail of the human CB_1_ receptor (Fig. 1) yielded single amplicons on agarose gel electrophoresis consistent with their expected sizes of 191 and 101 bp, respectively (Fig. 2A). Purified fragments were then inserted into the pCR-Blunt II-TOPO^™^ cloning plasmid. Once clones were checked by digestion with BamHI and BsrGI enzymes (Fig. 2B) and confirmed by sequencing, the inserts were subcloned into BamHI/NotI sites of the pGEX-6P1 multiple cloning site to generate bacterial expression plasmids encoding the recombinant fusion proteins GST-CB1_414–472_ and GST-CB1_414–442_ (Additional file 1: Fig. S1-2). After validating clones by sequencing (Additional file 2), plasmids were transferred to Rosetta^™^(DE3)pLysS strain for IPTG-induced recombinant protein expression.

### Analysis of the integrity and purity of synthetized GST fusion proteins

**Fig. 3:**
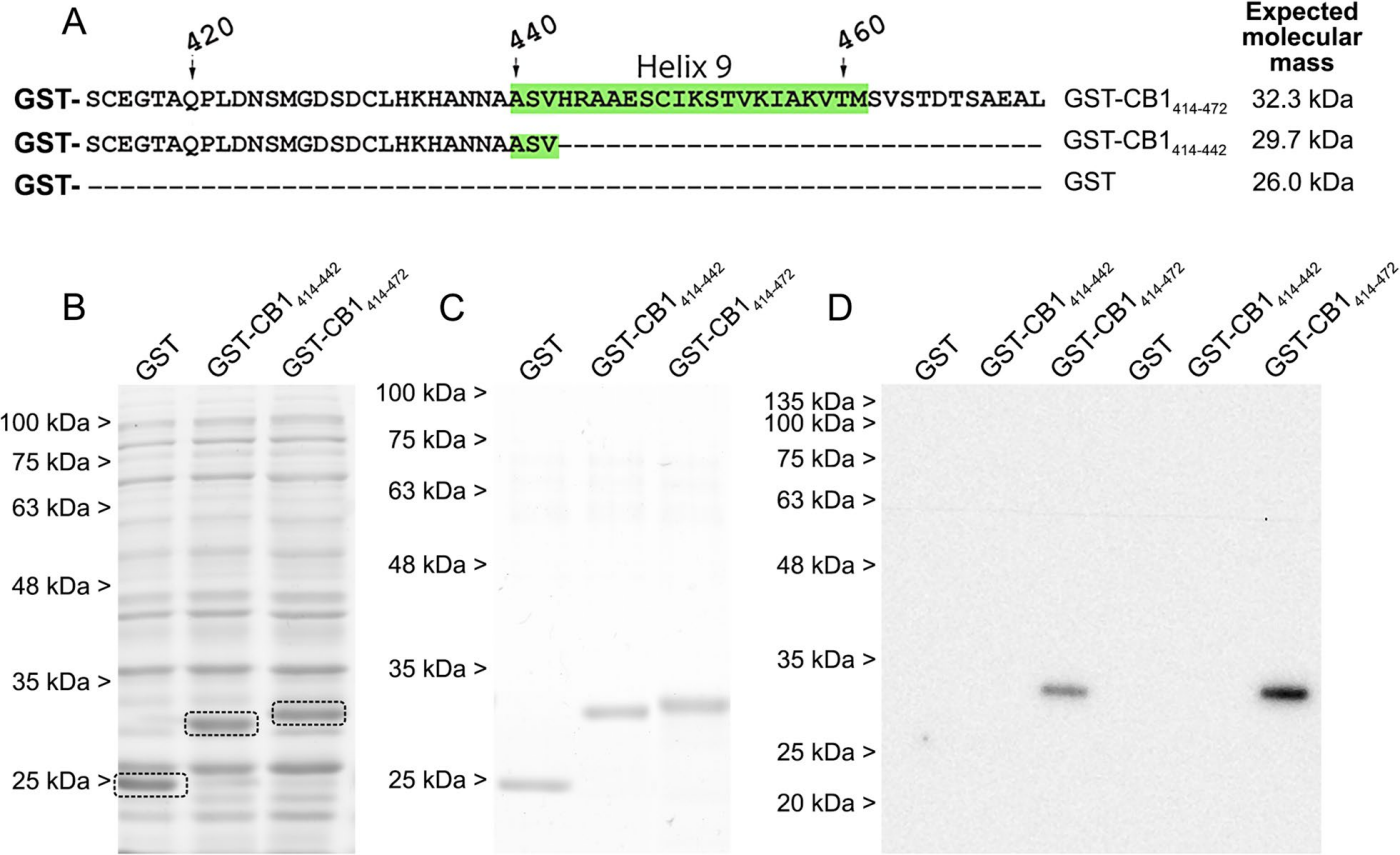
Analysis of the integrity of the GST-fusion proteins GST-CB1_414–442_ and GST-CB1_414–472_. **A** Linear representation of GST, GST-CB1_414–442_ and GST-CB1_414-472_ proteins and their theoretical molecular masses. **B** Analysis by SDS-PAGE and Coomassie blue staining of whole-cell pellet lysates from Rosetta^™^ (DE3) pLysS bacterial cultures induced by IPTG (3 h at 37 °C) for the expression of GST, GST-CB1_414–442_ and GST-CB1_414–472_ proteins. **C** Analysis by SDS-PAGE and Coomassie blue staining of GST, GST-CB1_414–442_ and GST-CB1_414–472_ proteins purified by affinity from the bacterial lysates shown in **B**. **D** Immunoblot of purified GST, GST-CB1_414–442_ and GST-CB1_414–472_ proteins using a rabbit polyclonal antibody raised against the 31 amino acid sequence at the C-terminus of the CB_1_ receptor. Only the GST-CB1_414–472_ construct carrying the antigen sequence was detected

The integrity of the fusion proteins GST-CB1_414–472_ and GST-CB1_414–442_ was analyzed by sodium dodecyl sulphate (SDS) polyacrylamide gel electrophoresis (SDS-PAGE) and Coomassie blue staining. In Coomassie blue stained lysates from whole bacterial pellets overexpressing GST, GST-CB1_414–442_ and GST-CB1_414–472_ proteins, extra bands consistent with the theoretical molecular masses of the corresponding constructs could be clearly observed (Fig. 3A, B). After affinity-purification, GST recombinant proteins migrated as single bands at their expected positions in SDS-PAGE gels (Fig. 3A, C), demonstrating the suitability of our strategy to produce highly purified GST-CB1_414–442_ and GST-CB1_414–472_ recombinant fusion proteins. Accordingly, the CB1-ImmGs rabbit polyclonal antibody (Table 1) raised against the carboxy-terminus (C-terminus) of the mouse CB_1_ receptor (Table 2) detected the purified GST-CB1_414–472_ protein containing the antigen sequence, but yielded no immunoreactivity against GST and GST-CB1_414–442_ proteins not carrying the antigen (Fig. 3A, D). Next, to optimize the induction conditions for the expression of the GST fusion proteins, we tested three variables (temperature, induction time, and presence or absence of glucose in the medium) known to affect the expression of recombinant proteins [39]. The results of these experiments showed that induction at 37 °C for 3 h in the absence of glucose was the best condition for the synthesis of GST-CB1_414–442_ and GST-CB1_414–472_ proteins (Additional file 1: Additional file results and Fig. S3 A, B).

**Table 1:** Primary polyclonal antibodies against CB_1_ receptor

Short name	Dilution	Host	Immunizing peptide	Source, Catalog N
CB1-Af380	0.2 µg/mL	Rabbit	Residues 443–473 of the mouse CB_1_ receptor	Frontier Institute Co., Ltd., CB1-Rb-Af380
CB1-Af450	0.4 µg/mL	Goat	Residues 443–473 of the mouse CB_1_ receptor	Frontier Institute Co., Ltd., CB1-Go-Af450
CB1-ImmGs	0.2 µg/mL	Rabbit	Residues 443–473 of the mouse CB_1_ receptor	ImmunoGenes Kft., Anti-CB1 polyclonal antibody

Antibody manufacturers: Frontier Science Co. Ltd., Hokkaido, Japan; ImmunoGenes Kft., Budakeszi, Hungary

**Table 2:** Sequence alignment of the C-terminal 31 residues of human, rat and mouse CB_1_ receptor

Orgainsm	NCBI Accession	Amino acid sequence
*Homo sapiens*	NP_001153698	442-VHRAAESCIKSTVKIAKVTMSVSTDTSAEAL-472
*Rattus norvegicus*	NP_036916	443-MHRAAESCIKSTVKIAKVTMSVSTDTSAEAL-473
*Mus musculus*	NP_031752	443-MHRAAESCIKSTVKIAKVTMSVSTDTSAEAL-473

### Stability over time and resistance to harsh denaturation of GST-CB1_414-442_ and GST-CB1_414-472_ constructs

**Fig. 4:**
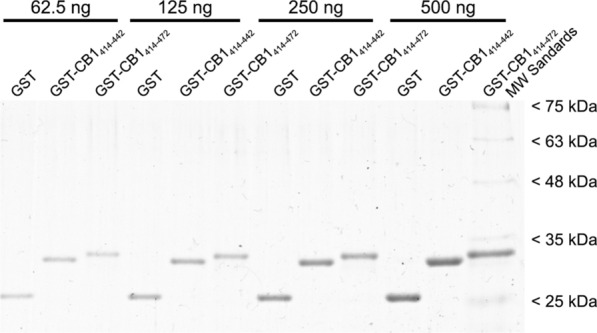
Analysis of the stability over time and of the resistance to strong denaturalization of GST fusion constructs. Increasing amounts of affinity purified GST, GST-CB1_414-442_ and GST-CB1_414-472_ proteins stored at −80 °C for more than one year and subjected to harsh denaturing conditions were analyzed by SDS-PAGE and Coomassie blue staining

Once the optimal conditions to produce GST-CB1_414-442_ and GST-CB1_414-472_ were established, we tested the resistance of these constructs to strong denaturing conditions and their stability over time. Because for still unclear reasons [40] some GST fusion proteins are degraded during commonly used strong/heavy denaturation conditions, GST fusion proteins were denatured by the ionic detergent SDS under non-reducing conditions and without heating in all SDS-PAGE assays described so far (see methods for details). To test the resistance of GST-CB1_414-472_ and GST-CB1_414-442_ proteins to harsh denaturation conditions and their stability over time, purified proteins that had been stored for more than 1 year at −80 °C were re-suspended in 2% SDS denaturing buffer containing 12% urea and 5% dithiothreitol (DTT) and heated at 95 °C during 5 min. As shown in Fig. 4, Coomassie blue stained GST-CB1_414-442_ and GST-CB1_414-472_ proteins subjected to such conditions and resolved by SDS-PAGE migrated according to their theoretical molecular masses, while no bands of higher or lower molecular masses were detected, indicating that storage at −80 °C for long periods and harsh denaturation conditions do not cause protein aggregation or degradation. Thus, our design produced stable protein products in aqueous solution over time.

### Estimation of CB_1_ receptor density in crude synaptosomes of the rat cerebral cortex by quantitative Western blot

**Fig. 5:**
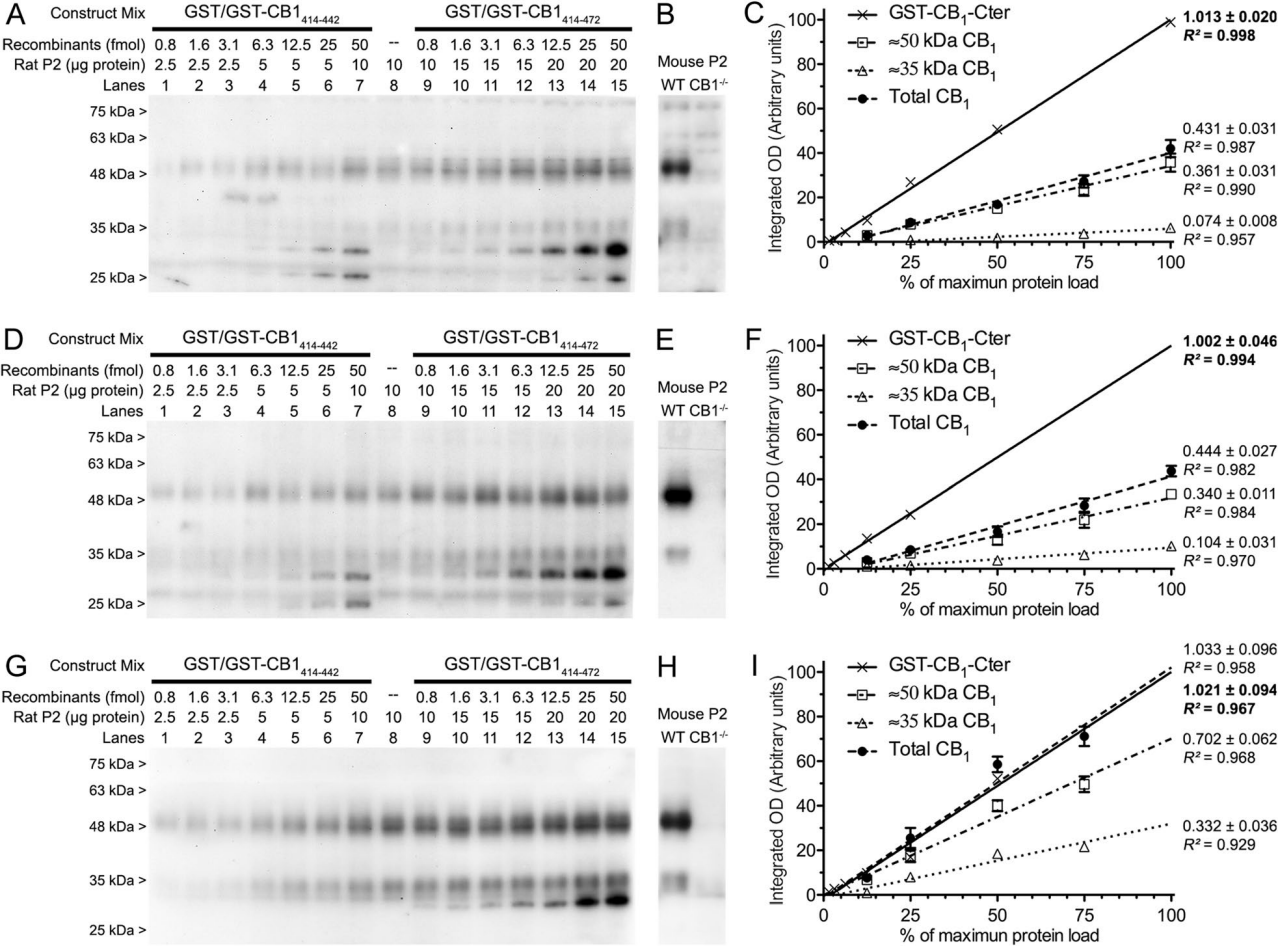
Quantitative Western blot analysis of CB_1_ receptor density on P2 membranes from rat brain cortex. **A**, **D**, **G** Increasing amounts of total protein from P2 membranes were loaded together with increasing molar amounts of combined GST/GST-CB1_414-442_ or GST/GST-CB1_414-472_ recombinant proteins, resolved side by side on SDS-PAGE and processed for immunoblotting using CB1-Af380 (**A**), CB1-Af450 (**D**) and CB1-ImmGs (**G**) polyclonal antibodies, all raised against the 31 amino acid sequence at the C-terminus of the CB_1_ receptor. **B**, **E**, **H** Immunoreactive bands profile detected in P2 samples of brain cortex from adult CB1-WT and CB1-KO mice were used to identify the CB_1_ receptor-specific signals detected by CB1-Af380 (**C**), CB1-Af450 (**F**) and CB1-ImmGs (**I**) antibodies. **C**, **F**, **I** Slopes obtained by linear regression analysis of the integrated optical density (Integrated OD) values of immunoreactivity corresponding to the endogenous CB_1_ receptor in P2 membranes and to the recombinant construct GST-CB1_414-472_. The integrated OD values were obtained by densitometric analysis of the immunoreactive bands produced by the endogenous CB_1_ receptor at ~ 50 and ~ 35 kDa and by the recombinant standard GST-CB1_414-472_ (subtracting the value of the non-specific signal produced by the GST-CB1_414-442_ protein) at increasing sample loads

CB1-Af380, CB1-Af450 and CB1-ImmGs antibodies (Table 1), all three raised against a peptide comprising the C-terminal 31 amino acids of the mouse CB_1_ receptor and highly conserved across human, mouse and rat (Table 2), were used for quantitative Western blot analysis of CB_1_ receptor density in rat cortical crude synaptosomes (P2 membranes), with purified GST-CB1_414-472_ (containing the immunogen sequence) and GST-CB1_414-442_ (without the immunogen sequence) constructs used as standard and negative control, respectively. Increasing amounts of P2 membranes (2–8 µg) together with combinations of purified GST and GST-CB1_414-472_ or GST and GST-CB1_414-442_ recombinant proteins (0.8–50 fmol) were resolved side by side on SDS-PAGE and detected by immunoblot using the aforementioned antibodies. The three antibodies recognized two bands migrating with apparent molecular masses of ~ 50 and ~ 35 kDa (Fig. 5A, D, G) that were CB_1_ receptor-specific, as they were detected in cortical membranes from wild type (CB_1_-WT) mice but not CB_1_ receptor null mutant (CB_1_-KO) mice (Fig. 5B, E, H). All three antibodies detected a single band consistent with the molecular mass of the GST-CB1_414-472_ protein (~ 32 kDa) within the full range of loading (0.8 and 50 fmol), although the signal corresponding to the lowest loading was difficult to distinguish from the background with CB1-Af380 and CB1-Af450 antibodies, while clearly stood out with the CB1-ImmGs antibody (Fig. 5A, D, G). Meanwhile, CB1-Af380 and CB1-Af450 antibodies clearly detected immunoreactive bands corresponding to the GST and GST-CB1_414-442_ proteins (both devoid of the antigenic sequence) at protein loads above 12.5 fmol (Fig. 5B, E), whereas CB1-ImmGs did not produce immunoreactivity at all for those recombinant proteins even at the highest loadings within the range (Fig. 5H). It is noteworthy that the non-specific signals produced by the CB1-Af380 and CB1-Af450 antibodies were of the same intensity for the GST and GST-CB1_414-442_ proteins, indicating that they resulted from IgG binding to the GST tag but not to the fragment comprising residues 414–442 of the cytosolic tail of CB_1_ receptor. These results evidenced that the three antibodies tested show a much higher affinity for epitopes within the immunogen compared to other peptide sequences. However, although all three antibodies showed high selectivity for their target, only the CB1-ImmGs antibody was strictly specific in our experimental paradigm.

Densitometric analysis of the immunoreactive bands corresponding to the endogenous CB_1_ receptor (~ 50 and ~ 35 kDa) and to the GST-CB1_414-472_ (~ 32 kDa) protein allowed us to obtain an estimate of the density of the endogenous CB_1_ receptor in crude synaptosomes of the rat cerebral cortex. Thus, the slopes obtained by linear regression analysis of the increasing values of integrated optical density (OD) corresponding to the immunoreactive signals of the endogenous CB_1_ receptor and of the GST-CB1_414-472_ protein (Additional file 1: Fig. S4 and Fig. 5C, F, I) were used to calculate an estimate of the molar amount of the ~ 50 and ~ 35 kDa CB_1_ receptor species per mass unit of P2 membrane protein (see methods for details). As shown in Table 3, the CB_1_ receptor density in P2 membranes of the rat cerebral cortex estimated with the CB1-Af380 and CB1-Af450 antibodies yielded similar values. Thus, the density corresponding to the ~ 50 kDa species was about 0.9 pmol/mg P2 protein, while the density of the ~ 35 kDa species was about 4 to 5 times lower. Consequently, no differences were observed between the two antibodies for the CB1 receptor density calculated for the sum of both signals (around 1.1 pmol/mg P2 protein). Notably, for both the ~ 50 kDa and ~ 35 kDa CB_1_ receptor species, the CB1-ImmGs antibody yielded statistically significantly higher CB_1_ receptor density values than the CB1-Af380 and CB1-Af450 antibodies. Thus, the estimated CB_1_ receptor density was approximately twice higher for the ~ 50 kDa CB_1_ receptor species with the CB1-ImmGs than with the CB1-Af380 and CB1-Af450 antibodies (around 1.7 versus 0.9 pmol/mg P2 protein, respectively) and, strikingly, four-fold higher for the ~ 35 kDa species (about 0.8 versus 0.2 pmol/mg P2 protein). Therefore, our results showed that CB1-ImmGs has a higher sensitivity than the other two antibodies tested, particularly in recognizing the ~ 35 kDa band, leading to a lower ratio of the ~ 50 kDa species over that of ~ 35 kDa (Table 3).

**Table 3:**
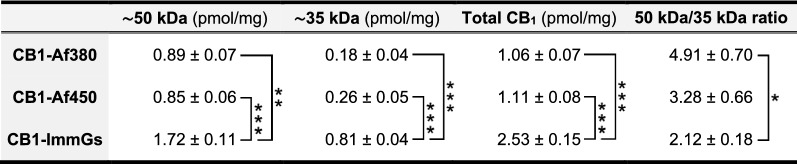
Results of quantitative Western blot analysis performed on P2 membranes of rat brain cortex

^*^p < 0.05, ** *p* < 0.01, *** *p* < 0.001, one-way ANOVA with Bonferroni post hoc test. Data are mean ± SEM (n = 3)

### Estimation of CB_1_ receptor density in crude synaptosomes of the rat cerebral cortex by radioligand saturation binding assays

The density of CB_1_ receptors in rat cerebral cortex P2 membranes was estimated by radioligand binding assays for comparison with results obtained by quantitative immunoblot using purified recombinant proteins as standard. Equilibrium binding saturation experiments were carried out using the high affinity CB_1_ receptor radioligands [^3^H]-SR141716A (selective CB_1_ receptor inverse agonist) and [^3^H]-CP55,940 (non-selective CB_1_ receptor full agonist), capable of detecting the inactive (G protein-uncoupled) and active (G protein-coupled) conformational states of the CB_1_ receptor, respectively.

**Fig. 6:**
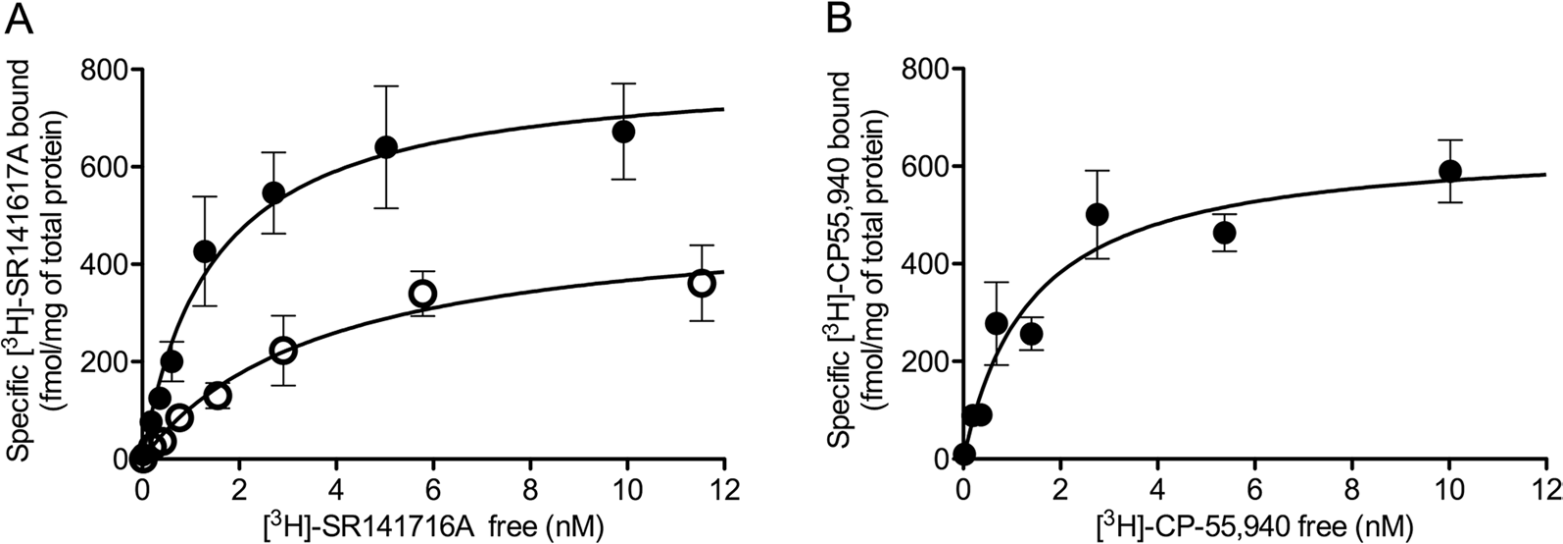
Radioligand binding assays in crude synaptosomes from rat brain cortex. **A** Representative saturation binding curves for [^3^H]SR141716A (0.01–10 nM) in the absence (empty circles) and in the presence of exogenously added 100 µM GTPγS (filled circles). **B** Representative saturation binding curve for [^3^H]-CP55940 (0.01–10 nM). Each point in curves corresponds to the mean ± SEM value of one independent experiment performed in triplicate. Mean values of the maximal number of sites (*B*_*max*_) and affinity (*K*_*D*_) averaged across three independent experiments are shown in Table 4

The specific binding of the radioligand [^3^H]-SR141716A in the range of 0.01–10 nM consisted of a saturable and high-affinity process (in nanomolar order), yielding experimental curves that were best fitted (by non-linear regression analysis) to a single population of sites with Hill coefficients close to unity. Saturation assays were performed both in the absence (control) and in the presence of the non-hydrolysable GTP derivative guanosine 5′-[γ-thio]triphosphate (GTPγS), which promotes GPCRs to switch to their inactive conformation of high affinity for inverse agonists. The analysis of the experimental saturation curve in the control condition resulted in a *B*_*max*_ value of 0.51 ± 0.01 pmol/mg protein for the [^3^H]-SR141716A ligand, and increased significantly to 0.81 ± 0.02 pmol/mg (39% increase, *p* < 0.001) upon addition of 100 µM GTPγS. Addition of 100 µM GTPγS also led to a significant increase in the affinity of [^3^H]-SR141716A binding with respect to the control condition (*p* < 0.05), with *K*_*D*_ values of 1.23 ± 0.13 nM and 3.39 ± 0.56 nM (mean ± SEM), respectively (Fig. 6A, Table 4).

**Table 4:**
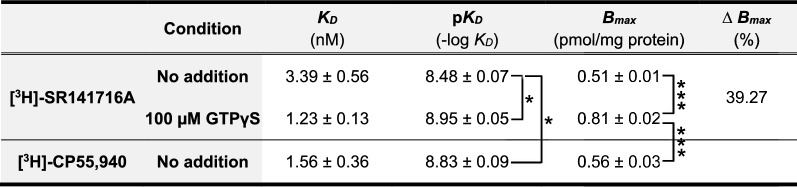
Results of radioligand saturation binding assays performed on P2 membranes of rat brain cortex

[^3^H]-SR141716A and [^3^H]-CP55,940 are inverse agonist and full agonist for the CB_1_ receptor, respectively. *B*_*max*_, maximum number of binding sites. *K*_*D*_, affinity. ∆*B*_*max*_, percent increase in *B*_*max*_ for radioligand [^3^H]-SR141716A after addition of 100 µM GTPγS relative to control (no addition) condition

*p < 0.05, *** *p* < 0.001, one-way ANOVA with Bonferroni post hoc test. Data are mean ± SEM of three independent experiments (n = 3)

The specific binding of the radioligand [^3^H]-CP55,940 in the range of 0.01–10 nM was also a saturable and high-affinity process, and experimental curves were again best fitted to a single population of sites, resulting in a *B*_*max*_ value of 0.56 ± 0.03 pmol/mg protein and a *K*_*D*_ value of 1.56 ± 0.36 nM (Fig. 6B, Table 4). Comparatively, the maximum number of binding sites (*B*_*max*_) was similar for [^3^H]-SR141716A and [^3^H]-CP55,940 radioligands when the binding assays were carried out in the absence of GTPγS (*p* = 0.59), but increased significantly for [^3^H]-SR141716A when the assay was performed in the presence of exogenously added GTPγS (*p* < 0.001). Thus, the non-selective CB_1_ receptor full agonist [^3^H]-CP55,940 recognized about 70% of the total CB_1_ receptor population relative to that estimated by the selective inverse agonist [^3^H]-SR141716A in the presence of GTPγS (Table 4).

## Discussion

During the last decades, there is a growing trend to replace radioligand-based techniques with more cost-effective and environmentally sustainable alternative procedures for the study of different aspects of G protein-coupled receptors (GPCR), including the cannabinoid CB_1_ receptor [41]. Thus, immunohistochemistry has largely displaced autoradiography for the study of CB_1_ receptor distribution in histological sections [42–44], with the advantage of providing more accurate information on receptor distribution [45]. In turn, Western blot has by far surpassed radioligand saturation binding techniques to study the changes in the expression of the CB_1_ receptor that occur during development [46, 47], after the administration of exogenous substances [48, 49] or in pathological conditions [20, 48, 50], among others. However, in most studies, the Western blot technique provides semi-quantitative data on the relative expression of the receptor against a reference sample, whereas the radioligand saturation binding technique yields fairly accurate estimates of the density of the receptor expressed as molar amount per unit mass of protein.

The above-described limitation of Western blot can be overcome by migrating in parallel polypeptides containing the immunogen of the antibody used and the biological sample under study, followed by densitometric analysis of the specific signals of both the polypeptide (used as a standard) and the biological sample. Indeed, during the last two decades, some studies have used protein standards to quantify the expression of different protein targets in tissues, cells and fluids [26–29, 51], although to our knowledge there are no studies that have applied this strategy to quantify GPCR density. Causes for this may lie in the difficulty of expressing recombinant GPCRs in bacterial hosts [52, 53] along with the low stability of recombinant GPCRs in aqueous solution. In the particular case of CB_1_ receptor, the full-length protein has been successfully produced in *E. coli* by inactivating the bacterial *DnaJ* chaperone [54]. However, this recombinant receptor may be of little use as a standard for analytical purposes due to its difficult purification and isolation from membrane components [55, 56] and to its hydrophobic nature that makes it prone to aggregation and unstable over time [32]. Yet, recombinant soluble fragments containing the antibody epitope are sufficient as standards for quantitative Western blot, thus overcoming these limitations. The C-terminal tail of CB_1_ receptor is especially suitable for such a purpose, since it is expected to be highly soluble as it is oriented towards the intracellular aqueous side of the cell membrane. In addition, the anti-CB_1_ antibodies designed against peptide sequences within this fragment are among the most selective and widely used ones [14, 38, 46, 48, 57–59]. Of these, the CB1-Af380 and CB1-Af450 antibodies used here, both raised against the 31 amino acid sequence at the C-terminus of the CB_1_ receptor, are among the most widely used in the last decade [46, 48, 57–59] and have been validated for immunohistochemistry and Western blot [60–62] in transgenic mice lacking the CB_1_ receptor. Moreover, we have recently provided robust data on the selectivity of these two antibodies for a variety of end uses including Western blot [30], and demonstrated the specificity of the third anti-CB_1_ antibody used here (CB1-ImmGs) for the analysis of CB_1_ receptor expression by Western blot [31]. Here again, using brain tissue from CB_1_-KO mice to dissect CB_1_ receptor-specific signals from non-specific ones, we identified with all three antibodies two CB_1_ receptor-specific bands in P2 samples of rat cerebral cortex that migrated with apparent molecular masses of ~ 50 and ~ 35 kDa on SDS-PAGE and corresponded to N-glycosylated and non-glycosylated receptor species, respectively, as we have recently shown [31].

Based on this background, we designed two DNA constructs to produce a pair of recombinant proteins useful as standard and negative controls for quantitative Western blot analysis of CB_1_ receptor levels in biological samples. One of them encoded residues 414 to 472 of the human CB_1_ receptor (CB1_414-472_), which comprises much of the cytosolic C-terminal tail of the receptor and includes the immunogen used to produce the CB1-Af380, CB1-Af450 and CB1-ImmGs antibodies, whereas the other one encoded the same fragment but with a truncation of the last 30 residues (CB1_414-472_) and, consequently, lacks the antigen. To allow purification by affinity and increase solubility [63], these constructs were inserted into the pGEX-6P1 downstream the GST gene for the inducible expression as GST fusion proteins GST-CB1_414-472_ and GST-CB1_414-442_. To further increase solubility, the cloned fragment of the C-terminal tail of the CB_1_ receptor was designed to lack the proximal hydrophobic helix 8 (residues 401–412) spanning the membrane-cytoplasm interface (Fig. 1) [64, 65]. With this strategy, we were able to produce highly purified GST-CB1_414-472_ and GST-CB1_414-442_ proteins for potential use as standard and negative control for quantitative Western blot analysis, respectively, and demonstrated their high solubility as well as their stability under harsh denaturing conditions and over time. Thus, after being stored at −80 °C in aqueous solution for more than one year and subjected to harsh denaturation, purified GST-CB1_414-472_ and GST-CB1_414-442_ proteins still migrated as a single band at their theoretical mass in SDS–PAGE gels and contained the expected primary sequences as demonstrated by immunoblot. Indeed, the three antibodies used were highly selective in detecting the GST-CB1_414-472_ fusion construct in the range of protein loadings from 0.8 to 6.2 fmol, whereas the GST-CB1_414-442_ construct used as a negative control was undetectable with the CB1-ImmGs antibody over the entire range between 0.8 and 50 fmol and weakly detectable with the CB1-Af380 and CB1-Af450 antibodies at loads greater than 6.2 fmol. Hence, we concluded that GST-CB1_414-472_ and GST-CB1_414-442_ fusion proteins met the criteria for which they were designed, namely their use as a standard and negative control for quantitative Western blot analysis of CB_1_ receptor density in P2 membranes of the rat cerebral cortex using well-characterized antibodies against 31 amino acids at the C-terminus of the CB_1_ receptor.

After validating antibodies and recombinant protein standards, we carried out quantitative Western blot to estimate CB_1_ receptor density in P2 samples of the adult rat cerebral cortex. The approach basically consisted in resolving side by side on SDS-PAGE increasing protein amounts of P2 samples and increasing molar amounts of GST-CB1_414-472_ and GST-CB1_414-442_ fusion proteins followed by immunoblot and densitometric analysis, and then comparing the intensity values of CB_1_ receptor-specific bands in P2 samples with those of the recombinant standard GST-CB1_414-472_ (subtracting from the latter the value of the signal corresponding to the negative control GST-CB1_414-442_). Despite its apparent ease, this approach has to meet the same requirements as any quantitative chemical analysis based on the use of selective ligands such as antibodies. Therefore, the experimental conditions must be set so that the magnitude of the change in the immunoreactive signals can be predicted as the amount of analyte varies. In practice, the relationship between the amount of sample loaded and the intensity of specific immunoreactivity must be linear for both the test sample and the standard sample [26–29]. For this, the amount of sample loaded and the dilution of the antibody are the critical experimental variables, which are also specific for each antibody and type of sample. Under our experimental conditions, regression analysis of the integrated OD values of the immunoreactive bands obtained with all the three antibodies revealed excellent linear dynamic ranges for both the endogenous CB_1_ receptor and the GST-CB1_414-472_ protein, with a minimum of four values within the linear range. Using the slope values of the resulting standard equations, the CB_1_ receptor density estimates obtained by quantitative Western blot with three different antibodies in P2 samples from adult rat cortex were of the same order of magnitude as those previously obtained using radioligand saturation assays [66, 67]. However, results also revealed marked differences among the three antibodies in their ability of to detect the entire CB_1_ receptor population in P2 membranes. Indeed, the sum of the densities of the CB_1_ receptor species migrating at ~ 50 and ~ 35 kDa was estimated to be more than double with the CB1-ImmGs (2.5 pmol/mg protein P2) than with the CB1-Af380 and CB1-Af450 antibodies (1.06 and 1.11 pmol/mg, respectively). These discrepancies may seem surprising, since all three antibodies were raised against the same immunogen, and are difficult to explain on the basis of intrinsic differences between the antibodies such as affinity for the antigen, which is known to vary by several orders of magnitude [68]. Thus, the binding strengths of the antibody to the endogenous CB_1_ receptor and to the recombinant standard are expected to be equally affected by affinity, resulting in equal ratios between endogenous CB_1_ receptor and recombinant standard signals for antibodies with different affinities. A more plausible explanation could arise from the effect that post-translational modifications in the sequence corresponding to the immunogen within the endogenous CB_1_ receptor could have on the affinity of the different antibodies. Indeed, phosphorylation at the C-terminus of the CB_1_ receptor after agonist binding and activation is known to be necessary for its proper internalization [69, 70], and six serine/threonine residues out of the 13 amino acids at the extreme C-terminus of the receptor have been identified as undergoing phosphorylation [71]. Consequently, the strength of binding to the antigen could result drastically reduced for antibodies with epitopes that include post-translationally phosphorylated residues. Because these post-translational modifications are catalyzed by eukaryotic enzymes known as G protein-coupled receptor kinases, they only occur in the sequence of the endogenous CB_1_ receptor of the P2 samples but not in that of the recombinant protein GST-CB1_414-472_ used as a reference standard. Therefore, the estimations of CB_1_ receptor density obtained with these antibodies would be biased downwards due to their inability to detect a subpopulation of endogenous CB_1_ receptors with no impact in their capacity to recognize the recombinant standard. Consistent with this idea, it is conceivable that the IgGs of the CB1-Af380 and CB1-Af450 antibodies target epitopes that include modified residues to a greater extent than the CB1-ImmGs antibody, leading to a less biased estimate in the case of the latter. More intriguingly, the decreased ability of CB1-Af380 and CB1-Af450 antibodies relative to CB1-ImmGs to detect the entire population of CB_1_ receptors in P2 samples of the adult rat cortex was more marked for the non-glycosylated than for the N-glycosylated receptor species migrating at ~ 35 kDa and ~ 50 kDa, respectively. Thus, compared to CB1-Af380 and CB1-Af450 antibodies, CB1-ImmGs detected approximately twice the density of glycosylated CB_1_ receptors but three to four times the density of non-glycosylated ones. Because the two asparagines involved in the N-glycosylation of the CB1 receptor are located at the amino terminus of its primary sequence and the Western blot technique is performed under denaturing conditions, it is extremely unlikely that the glycosylation state of the receptor could alter the binding of the antibody to the antigen located at the C-terminus. In line with the above argument of a variable effect of antigen phosphorylation on the affinity of the different anti-CB_1_ antibodies, is tempting to speculate that the lower ability of the CB1-Af380 and CB1-Af450 antibodies to detect the non-glycosylated CB_1_ receptor species compared to the N-glycosylated one could be due to a higher degree of phosphorylation of the former. Consistent with this, a recent report revealed that a mouse CB_1_ receptor splice variant lacking N-glycosylation sites shows a marked increase in the kinetics and rate of internalization upon stimulation with the agonist WIN55,212-2 [72]. Future studies aimed at mapping the preferential binding site of each antibody within the C-terminal 31 amino acids of the CB1 receptor, in combination with in vitro phosphorylation/dephosphorylation assays, will be necessary to address these questions. In any case, our results point to CB1-ImmGs as one of the most specific and suitable antibodies for determining the density of CB_1_ receptors in biological samples, and emphasize the importance of characterizing by means of analytical techniques those antibodies intended for quantitative and semi-quantitative purposes.

As aforementioned, the CB_1_ receptor levels estimated here by quantitative Western blot on P2 membranes from adult rat cortex are in good agreement with previously reported values as determined by radioligand saturation binding assays on similar samples [66, 67]. Nonetheless, for an accurate comparison between both methods, we estimated CB_1_ receptor density by radioligand saturation binding on the same P2 membranes used here for quantitative Western blot. It is important to point out that the radioligand chosen for these assays may constrain the possibility to detect the entire receptor population, as the number of sites to which GPCR ligands bind depends on their pharmacological properties, specifically their efficacy as agonist, antagonist or inverse agonist [5, 6]. Thus, agonists and inverse agonists bind active and inactive conformational states of GPCRs, respectively, while neutral antagonists show no preference and are therefore the only ligands that can provide a measure of the entire receptor population [73]. However, inverse agonists in combination with high concentrations of guanine nucleotides, are a good alternative for this purpose when radiolabeled neutral antagonists are not available. According to the ternary complex model of GPCR signaling, addition of the non-hydrolysable GTP derivative GTPγS in saturation binding assays causes dissociation of the G protein-GPCR complexes, rendering receptors to switch to their inactive state of high affinity for inverse agonists [7, 8, 74]. Since there are no radioactively labeled neutral antagonists for the CB_1_ receptor in the market, we used the selective CB_1_ inverse agonist [^3^H]-SR141716A as a radioligand for saturation binding assays. Initially considered to be a neutral antagonist, [^3^H]-SR141716A has been used directly to measure the total number of CB_1_ receptor binding sites [75, 76]. However, SR141716A has been classified as an inverse agonist based on experimental evidence denoting its efficacy in reversing constitutive receptor signaling in the absence of cannabinoid agonists [74, 77–81]. Here, radioligand saturation binding assays using [^3^H]-SR141716A were performed in the absence and presence of 100 μM GTPγS. Indicative of the selectivity of [^3^H]-SR141716A for the G protein-uncoupled inactive conformation of CB_1_ receptors, the addition of GTPγS led to a statistically significant 39% increase in the maximum number of [^3^H]-SR141716A binding sites in P2 samples of the adult rat cortex. Therefore, our results further support the well-established behavior of SR141716A as an inverse agonist at CB_1_ receptors and fit well with the predictions of the ternary complex model of GPCR signaling [7, 8]. In addition, radioligand binding assays were carried out using the CB_1_ receptor agonist [^3^H]-CP55,940, which exclusively recognize CB_1_ receptors in active conformation. Accordingly, [^3^H]-CP55,940 did not reach the maximum number of binding sites labeled by [^3^H]-SR141716A in the presence of GTPγS, which reinforces the suitability of our experimental approach. Nonetheless, the sum of the maximum number of sites labeled by [^3^H]-SR141716A in the absence of GTPγS (0.51 pmol/mg P2 protein) and by the CB_1_ receptor agonist [^3^H]-CP55,940 (0.56 pmol/mg P2 protein) exceeded by far the maximum number of binding sites of [^3^H]-SR141716A in the presence of GTPγS (0.81 pmol/mg P2 protein). This can be easily explained by the fact that agonists and inverse agonists bias the equilibrium of the system towards more active and inactive conformational states of the GPCRs, respectively [7, 8]. In conclusion, it is conceivable that the CB_1_ receptor density value obtained by saturation binding experiments using [^3^H]-SR141716A under conditions in which CB_1_ receptors were forced to their inactive conformation by the addition of GTPγS represents a good estimate of the entire receptor population.

Focusing on the nuclear theme of our study, the CB_1_ receptor density estimated by quantitative Western blot was of the same order of magnitude as that obtained by the radioligand saturation binding technique. However, despite the overall consistency of results, quantitative Western blot yielded a maximal estimate of about three times the value obtained by radioligand saturation binding (2.53 and 0.81 pmol/mg P2 protein, respectively). The bias that the specific factors inherent to each of the techniques can imprint on the final result could largely explain this difference. For instance, antibodies have complete access to antigen under the denaturing conditions used for Western blotting, whereas the native environment of cell membranes used in saturation binding assays could reduce receptor occupancy by hampering the ligand's ability to bind receptors in difficult-to-reach subcellular compartments. In this sense, considering that N-glycosylation has been consistently linked to the correct transport and stabilization of GPCRs on the cell surface [82] and that non-glycosylated CB_1_ receptors show a higher rate of stimulation-induced internalization [72], it is tempting to speculate that the ~ 35 kDa non-glycosylated CB_1_ receptor species detected by Western blotting is less expressed on the cell surface of P2 membranes and thus less accessible to radioligands than the ~ 50 kDa N-glycosylated species. In addition, during the isolation procedure of P2 membranes, part of the plasma membrane vesicles formed upon cell lysis could be made so that the inner leaflet of the phospholipid bilayer faces outward, leaving the ligand-binding pocket internally oriented and less accessible to the radioligand.

Apart from physical factors that can interfere with the law of mass action in antigen-antibody and ligand-GPCR binding, the different nature of these interactions and the potential factors that can affect their strength are additional sources of discrepancy between the results obtained by quantitative Western blot and radioligand saturation binding techniques. Thus, the affinity of the antibody for its antigen is not conditioned by conformational changes of the interacting elements, whereas the affinity of ligands depends largely on the ligand type and conformational state of GPCRs [7, 8, 82]. For this reason, radioligand saturation binding assays were performed in the presence of GTPγS to shift the conformational equilibrium of CB_1_ receptors toward an inactive state with high affinity for the inverse agonist [^3^H]-SR141716A, thus minimizing the impact of conformational bias. This experimental approach is based on the theoretical framework of the ternary complex of GPCR signaling, with the ligand, receptor and G proteins as the key elements of the system (see discussion above). Yet, it cannot be completely ruled out that intracellular transducers other than G proteins may form complexes with GPCRs, stabilizing part of the receptor population in a conformation resistant to the effects of GTPγS, with high and low affinities for agonists and inverse agonists, respectively. In this sense, β-arrestin has been pointed out as a major GPCR-interacting transducer that could form an alternative ternary complex with the receptor in a high-affinity agonist binding conformation. Moreover, unlike agonist-receptor-G protein complexes, agonist-receptor-β-arrestin complexes are relatively stable and insensitive to nucleotides and ions [83]. Under the assumption that the described phenomenon is universal among GPCRs and considering the large body of evidence supporting that CB_1_ receptors and β-arrestin interact [69, 70, 84–87], it is conceivable that CB_1_ receptor-β-arrestin complexes form in the P2 membranes used in saturation radioligand binding assays. Consequently, a subpopulation of CB_1_ receptors could be insensitive to GTPγS as a result of their stabilization in the active conformation with low affinity for the inverse agonist [^3^H]-SR141716A, leading to an underestimation of the number of CB_1_ receptors.

Additional technical and methodological issues could explain the discrepancies between the two methods. Among these, non-specific ligand binding makes it difficult to obtain quality radioligand binding parameter data in saturation binding assays [1–3]; and this drawback may be particularly relevant for lipophilic compounds such as CB_1_ receptor ligands. Although we achieved acceptable specific binding levels above 70% of total binding for both [^3^H]-SR141716A and [^3^H]-CP55,940 radioligands, the estimates of binding parameters may still be slightly distorted. Also worthy of consideration is that the estimated value of the maximum number of binding sites (*B*_*max*_) corresponds to the asymptotic plateau of the saturation curve obtained by non-linear regression fit of the binding values measured experimentally at increasing concentrations of the radioligand. Since this plateau is only reached at non-experimental high radioligand concentrations, the obtained *B*_*max*_ value may differ slightly from the real one. A technical issue that could interfere with estimation of CB_1_ receptor density by quantitative Western blotting results from the possible presence of contaminating bacterial proteins in purified recombinant standard samples. Although affinity purification of GST fusion proteins using glutathione immobilized on magnetic agarose beads is highly efficient, the presence of bacterial protein remnants non-specifically bound to the beads during the purification process cannot be completely ruled out. Therefore, it is possible that small amounts of bacterial proteins distributed over a wide range of molecular masses, but below the threshold of detection by Coomassie blue staining, may together contribute to an overestimate of the amount of purified protein measured by the Bradford method. Consequently, the slope value calculated for the recombinant standard would be lower than the real one, which would lead to an overestimation of the density of the CB_1_ receptor in the P2 samples.

## Conclusions

Radioligand saturation binding and quantitative Western blot techniques used for determination of CB_1_ receptor density in brain membranes provided roughly similar results, but with differences consistent with the theoretical underpinnings of each technique. Therefore, rather than invalidating the suitability of one or another technique for the intended purpose, the observed discrepancies emphasize the importance of interpreting the results considering the theoretical and methodological bases of each of the techniques, which ultimately determine their advantages and limitations. Thus, we demonstrate here the suitability of our design based on the use of GST fusion proteins as standards to quantitatively determine the density of CB_1_ receptors by Western blot, an approach that could theoretically be applied to any biological sample as well as to any GPCR for which specific antibodies are available. It is important to highlight that this methodology makes it possible to quantitatively determine the expression of GPCRs in a variety of preparations and conditions in which the radioligand saturation technique is not applicable. For example, when the amount of sample is limiting, quantitative Western blot is a good option as it requires much less amount than radioligand binding techniques. Likewise, when the conditions of storage or sample collection alter the native conformation of the receptor, the use of Western blot (but not the binding of radioligands) is possible. Solubilization is also incompatible with the filtration step used in saturation radioligand binding assays to separate bound from free radioligands, even when using non-ionic detergents that maintain the native conformation of the receptor. Furthermore, quantitative Western blot solves several important limitations inherent to the radioligand saturation binding approach such as health risks, generation of radioactive waste, or the need for well-controlled work environments and highly qualified technical personnel. In addition, the production of recombinant fusion proteins is rapid, simple, and cost-effective, making quantitative Western blot readily accessible to basic research laboratories as an alternative to radioligand binding techniques for quantification of CB_1_ receptors and other GPCRs in biological samples.

## Methods

### Chemicals and reagents

Guanosine 5′-[γ-thio]triphosphate (GTPγS) tetralithium salt (Cat. G8634; Sigma-Aldrich, Madrid, Spain); [^3^H]-CP55,940 (Cat. NET1051025UC; Specific activity, 139.6 Ci/mmol; Perkin-Elmer, Madrid, Spain); [^3^H]-SR141716A (Cat. NET1158250UC; Specific activity, 43 Ci/mmol, Perkin-Elmer); *(R)*-( +)-WIN 55,212-2 mesylate salt (Cat. W102; Sigma-Aldrich); Protease Inhibitor Cocktail (Cat. 539134; Sigma-Aldrich); Bovine Serum Albumin Fraction V, fatty acid free (Cat. 10775835001; Sigma-Aldrich).

### Molecular cloning

The DNA sequences coding for most of the cytosolic tail of the human CB_1_ receptor, from serine 414 to the terminal leucine 472 (CB1_414–472_,) or for the same sequence truncated of the last 30 (CB1_414-442_) residues (Fig. 1), were amplified by PCR using Fw and Rv primer pairs carrying BamHI and NotI restriction sites at their 5' ends (Table 5) for cloning in frame with the upstream glutathione S-transferase (GST) tag of the bacterial expression vector pGEX-6P1 (Cat. GE28-9546-48; Sigma-Aldrich). The purified PCR products were inserted into the pCR-Blunt II-TOPO^™^ cloning plasmid using the Zero Blunt^™^ TOPO^™^ cloning kit (Cat. K280002; Thermo Fisher Scientific, Barcelona, Spain). TOPO clones containing the insert with the correct sequence were selected for digestion-ligation cloning into the bacterial expression vector pGEX-6P1, which allows expression of GST and GST-tagged constructs under the control of an isopropyl β-d-thiogalactoside (IPTG) inducible tac promoter. The sequencing-verified pGEX-P1 plasmids (Additional file 2) carrying the coding sequences for the polypeptides CB1_414–472_ and CB1_414–442_ fused to the C terminus of GST (GST-CB1_414–472_ and GST-CB1_414–442_) and the empty pGEX-P1 plasmid were transferred by heat shock to the BL21-derived bacterial strain Rosetta^™^(DE3)pLysS (Cat. 70956; Sigma-Aldrich) for GST protein production (Additional file 1: Additional file methods).

**Table 5:** Primers used for PCR amplification of DNA encoding fragments of human CB_1_ receptor cytosolic tail

Encoded peptide	Forward primers (5′- > 3′)	Reverse primers (5′- > 3′)
CB1_414–472_	**G^GATC**CTGTGAAGGCACTGCGCAGC	**GC^GGCCGC**TCACAGAGCCTCGGCAGACGTGTC
CB1_414–442_	**G^GATC**CTGTGAAGGCACTGCGCAGC	**GC^GGCCGC**TCAAACACTGGCTGCATTGTTTGC

BamHI and NotI restriction sites are highlighted in bold. The ^ symbols indicate the cleavage sites for restriction enzymes. Underlined TCA sequences represent translation stop sites

### Production and purification of GST fusion proteins

Pre-cultures of transformed Rosetta clones were inoculated to an OD_600_ of 0.1 in 50 mL LB Broth containing ampicillin and chloramphenicol and grown at 37 °C with shaking at 200 rpm until an OD_600_ of 0.5–0.6 was reached. To induce the expression of the GST-CB1_414–472_ and GST-CB1_414–442_ fusion proteins, IPTG (Cat. 10724815001; Sigma-Aldrich) was added to a final concentration of 0.1 mM along with half the initial dose of ampicillin. Unless stated otherwise, cultures were incubated for an additional three hours at 37 °C and 200 rpm followed by centrifugation at 3500 × g for 20 min. The resulting bacterial pellet was stored at −80 °C for subsequent lysing and purification of the recombinant GST-fusion proteins. 1 mL of each culture was centrifuged apart for subsequent analysis of the bacterial pellet by SDS-PAGE and Coomassie blue staining. GST fusion proteins were purified using Pierce™ Glutathione Magnetic Agarose Beads (Cat. 78602; Thermo Fisher Scientific) following the procedure recommended by the supplier (Additional file 1: Additional file methods). The amount of protein was estimated by the microplate Bradford method, using the Protein Assay Dye Reagent Concentrate (Cat. 500-0006; Bio-Rad, Madrid, Spain) and bovine γ-globulin (Cat. 500-0208; Bio-Rad) as standard. Bradford assay-based estimations were re-adjusted by densitometric scanning of Coomassie blue-stained bands on SDS-PAGE gels, using integrated OD of purified recombinant GST as reference.

### Preparation of crude synaptosomes rat brain cortical membranes of the rat cerebral cortex

P2 membranes from the adult rat brain cortex were obtained as previously reported [30, 88]. Briefly, frozen brain cortices from five adult male Sprague–Dawley rats (225–250 g) from SGIker facilities of the University of the Basque Country (UPV/EHU, Spain) were thawed in ice cold Tris/Sucrose buffer (20 mM Tris-HCl, 1 mM EGTA, 0.32 M sucrose, pH 7.4) containing protease inhibitors (1 mM phenylmethylsulfonyl fluoride and 0.5 mM iodoacetamide) and then homogenized in 10 volumes of the same buffer using a teflon glass homogenizer. Once a homogeneous suspension was obtained, the whole homogenate was centrifuged in conical tubes at 1100 × g for 10 min at 4 °C in a high-speed centrifuge (Kontron, Centrikon T-42 K). The sediment (pellet) was discarded and the supernatant was centrifuged again at 40,000 × g for 10 min. The obtained pellet was resuspended in the original volume of Tris/Sucrose buffer and centrifuged again. After repeating this procedure, the pellet was resuspended in the same volume and distributed into in 1.5 mL Eppendorf tubes (1 mL/tube) and subjected to an additional centrifugation at 40,000 × g for 10 min. The supernatants were carefully aspirated without disturbing the final pellets (P2 membrane fraction), which were stored at −80 °C until use. The amount of protein was determined in one of the pellets as described above. Four CB_1_-WT and four CB_1_-KO mice, kindly provided by Dr. Giovanni Marsicano (Institute François Magendie, Bordeaux, France), were used to prepare P2 membranes identically as described above. All experiments were approved by the Committee of Ethics for Animal Welfare of the University of the Basque Country (UPV/EHU; CEBA/146/2010 and CEBA/61/2010) and conducted following guidelines of the Directive of the European Commission (2010/63/EU) and Spanish regulations (RD 53/2013) for care and management of experimental animals.

### SDS-PAGE and Western blot

Western blot studies were performed as previously reported with minor modifications [88–90]. Briefly, known amounts of total protein from P2 fractions were heated for 5 min at 60 °C in urea-denaturing buffer (20 mM Tris-HCl, pH 8.0, 12% glycerol, 12% urea, 5% DTT, 2% SDS, 0.01% bromophenol blue), whereas recombinant fusion proteins were denatured by the ionic detergent SDS (2%, w/v) under non-reducing conditions and without heating, unless otherwise indicated. These conditions were assumed to be sufficient for denaturation of the constructs, as they were expected to be highly hydrophilic and contain no disulfide bonds as predicted by the UniProt database (GST ID: P08515; CB_1_ ID: P21554) for both the GST protein and any of the CB_1_ receptor fragments fused to GST. Increasing amounts of P2 protein (2.5, 5, 10, 15, and 20 µg) from three different aliquots were loaded side by side on the same gel along with known amounts (0.78125, 1.5625, 3.125, 6.25, 12.5, 25 and 50 fmol) of the recombinant protein GST-CB1_414-472_ (containing the antigen for the antibodies used for quantitative determination of CB_1_ receptor density) and of the proteins GST and GST-CB1_414-442_ (devoid of the antigenic sequence). Proteins were resolved by electrophoresis in 12% SDS-PAGE gels using the Mini Protean II gel apparatus (Bio-Rad; Hercules, CA, USA). Subsequently, gels were stained with Coomassie blue dye or proteins were transferred to polyvinylidene fluoride (PVDF) membranes (Amersham Biosciences, Piscataway, NJ, USA) at 30 V overnight at 4 °C using the Mini TransBlot transfer unit (Bio-Rad; Hercules, CA, USA) and processed for immunoblot analysis. Blots were blocked at 20–25 °C for 1 h in blocking solution (0.2 M phosphate-buffered saline pH 7.4 -PBS-, containing 5% non-fat dry milk -Cat. 1,706,404; Bio-Rad-, 0.5% bovine serum albumin -BSA, Sigma-Aldrich-, 0.2% Tween-20 -Sigma-Aldrich-), followed by overnight incubation at 4 °C with immunogen affinity purified anti-CB_1_ antibodies CB1-Af380, CB1-Af450 and CB1-ImmGs diluted in blocking solution without milk (Table 1 for details). After three washes (10 min each) at 20–25 °C with PBS containing 0.1% Tween-20, blots were incubated for 2 h at 20–25 °C with horseradish peroxidase conjugated rabbit anti-goat IgG (A5420; Sigma-Aldrich) or horseradish peroxidase conjugated donkey anti-rabbit IgG (NA934; Amersham Biosciences) secondary antibodies, all diluted to 1:10,000 in blocking solution. After three additional washes as above, immunoreactive bands were visualized with Clarity Western ECL Substrate (#1705061; Bio-Rad) according to the manufacturer instructions. A color pre-stained broad-range protein ladder (MB090, NZYtech, Lisbon, Portugal) was used to estimate the molecular mass of individual bands.

### Radioligand binding assays

Briefly, all saturation binding experiments were carried out under shaking for 90 min at 30 °C in a thermostatic bath in a final volume of 1 mL per tube. The incubation medium was 50 mM Tris-HCl buffer containing 3 mM MgCl_2_, 0.2 mM EGTA, 5 mg/mL fatty acid-free BSA (Sigma-Aldrich), pH 7.4. Bound and free radioligand were separated by rapid filtration (Harvester, Brandel), followed by extensive washing with cold buffer (2 × 4 mL) through Whatman GF/C glass fibre filters, prewetted for 2 h with 2% SDS in order to negatively charge the filters and decrease the non-specific binding of the radioligands to the filters. Individual filters with retained radioligand-receptor complexes were placed in vials and 4 ml of OptiPhase HiSafe 2 scintillation fluid (Perkin Elmer) was added per vial and kept in the dark for 2 h. Radioactivity was then measured by liquid scintillation spectrophotometry (Packard 1600 TR, Tri-Carb). To determine non-specific binding, WIN55212-2 was added at a concentration of 10 µM. To fix the optimal amount of total protein from the P2 fractions, experiments were performed in the range of 15 to 500 µg total protein/tube with a concentration of 0.5 nM [^3^H]-CP55940 or 1.5 nM [^3^H]-SR141716A. For both radioligands, a concentration of 100 µg of total protein provided specific binding levels higher than 70% of the total binding. For saturation experiments, 8 concentrations in the range of 0.015–10 nM were assayed. In the case of the [^3^H]-SR141716A specific binding, the effect of guanine nucleotides on the affinity constant (*K*_*D*_) and maximal density of sites (*B*_*max*_) parameters was studied by adding a 100 µM concentration of the non-hydrolysable GTP analogue GTPγS.

### Data analysis

For the quantitative analysis of the density of the CB_1_ receptor by Western blot, the integrated OD of the specific immunoreactive bands corresponding to the endogenous CB_1_ receptor in P2 membranes of the rat cerebral cortex and to the antigen-containing GST-CB1_414-472_ fusion protein was measured using Fiji-ImageJ 1.53f51 (NIH, Bethesda, MA, USA). In addition, we measured the OD corresponding to the non-specific signal produced by the GST-CB1_414-442_ protein, which is devoid of the antigenic sequence. These values were subtracted from the corresponding OD values produced by GST-CB1_414–472_ protein. Then, standard equations were generated by linear regression analysis for the CB_1_-immunoreactive signals at ~ 50 kDa CB_1_, ~ 35 kDa and total CB_1_ (sum of ~ 50 and ~ 35 kDa signals) corresponding to endogenous CB_1_ receptor in P2 samples and for the immunoreactive signals at ~ 32 kDa produced by increasing molar amounts of GST-CB1_414-472_ recombinant protein. Values corresponding to saturated signals that were outside the linear range were excluded from the analysis and at least four points were used for linear regression curve fitting (Additional file 1: Fig S4). The resulting linear regression curve equation of GST-CB1_414-472_ protein was used to normalize all OD data, which were expressed as the percentage relative to the theoretical OD resulting from the maximum load of GST-CB1_414-472_. For each of the three P2 samples run side by side with the GST-CB1_414-472_ recombinant protein, CB_1_ receptor density values were obtained for the ~ 35 kDa CB_1_ and ~ 50 kDa CB_1_ species and for the total CB_1_ signal (Fig S4) using the equation $\frac{{\left( {\frac{ME}{{MR}}} \right) * 50 fmol}}{20 \mu g}$, where *M*_*E*_ and *M*_*R*_ are the slopes corresponding to the signals produced by the endogenous CB_1_ receptor proteins in P2 membranes and by the GST-CB1_414-472_ recombinant protein, respectively. CB_1_ receptor density values were obtained as the mean ± SEM of the values obtained from the three P2 samples (n = 3) resolved side by side on SDS-PAGE together with the GST-CB1_414-472_ and GST-CB1_414-442_ recombinant proteins. Standard deviation values for the $\sim 35\mathrm{ kDa CB}1/\mathrm{Total CB}1$ and $\sim 50\mathrm{ kDa CB}1/\mathrm{Total CB}1$ ratios were determined via Taylor expansion using the formula $\left| {\Delta RAB} \right|$= $RAB \cdot \sqrt {\left( {\frac{\Delta A}{A}} \right)^{2} + \left( {\frac{\Delta B}{B}} \right)^{2} }$, where $\Delta RAB$ is the standard deviation of the ratio of slope A over slope and $\Delta A$ and $\Delta B$ the standard deviations of slopes A and B.

To analyze the results of saturation binding assays, a computerized iterative procedure in GraphPad Prism was used to directly fit the experimental data to equations modelling one and two binding sites. The statistical differences between one- or two-site models for curve fitting to saturation data were determined by comparing the residual variance between the actual and predicted data points from both fittings, and the *F* statistic was computed as described by Munson and Rodbard [91]. For the statistical analysis of the differences between the affinity constants, the *K*_*D*_ values were logarithmically transformed, since it has been shown that parameters such as *K*_*D*_ and *EC*_*50*_ obtained experimentally have a log-normal distribution and, therefore, the statistical analysis should be performed as such [92, 93]. In contrast, comparisons between *B*_*max*_ values were made directly from the values expressed as mean ± SEM, as they can be assumed to have a normal distribution [94].

All data were organized and statistically analyzed using GraphPad Prism version 5.0 (GraphPad Software Inc., San Diego, CA, USA). The statistical significance of differences between the means were analyzed by one-way ANOVA followed by Bonferroni’s post hoc test for pairwise comparison with GraphPad Prism 5.0 software considering a confidence level of 95% (*p* < 0.05).

## Supplementary Information

**Additional file1**: **Additional file results.** Optimization of conditions for induction of GST fusion protein expression.** Additional file methods**. Molecular cloning. Production and purification of GST fusion proteins. **Figure S1.** Complementary DNA encoding GST-CB1_414-472_ fusion protein and primary amino acid sequence thereof. **Figure S2.** Complementary DNA (uppercase letters) encoding GST-CB1_414-442_ fusion protein and primary amino acid sequence thereof. **Figure S3.** Optimization of the conditions for the IPTG-inducible expression of fusion proteins GST, GST-CB1_414-442_, and GST-CB1_414-472_. **Figure S4.** Results of quantitative Western blot analysis of CB_1_ receptor density obtained on each of three P2 membrane samples from rat cerebral cortex tested with CB1-Af380, CB1-Af450 and CB1-ImmGs antibodies.

**Additional file2**: zip-compressed files of [i] theoretical sequences corresponding to pGEX-6P1 plasmids with DNA constructs inserted downstream the GST gene for the inducible expression of GST fusion proteins GST-CB1_414-472_ and GST-CB1_414-442_ and [ii] results of sequencing

## Data Availability

All data for this study are included in this published article and its additional files. Raw data used for statistics are available on reasonable request.

## Abbreviations

B_max_: Maximum number of binding sites
C-terminal: Carboxy-terminal
C-terminus: Carboxy-terminus
CB_1_ receptor: Cannabinoid receptor type 1
CB_1_-KO: CB_1_ receptor null mutant
CB_1_-WT: CB_1_ receptor wild type
DTT: Dithiothreitol
GPCR: G protein-coupled receptor
GST: Glutathione S-transferase
GTPγS: Guanosine 5′-[γ-thio]triphosphate
IPTG: Isopropyl β-_D_-1-thiogalactopyranoside
K_D_: Affinity
OD: Optical density
P2 membranes: Crude synaptosomes
SDS-PAGE: SDS polyacrylamide gel electrophoresis
SDS: Sodium dodecyl sulphate
