# Supplementary material for: Design and validation of recombinant protein standards for quantitative Western blot analysis of cannabinoid CB1 receptor density in cell membranes: an alternative to radioligand binding methods
Source: Microb Cell Fact. 2022 Sep 15;21:192. doi: 10.1186/s12934-022-01914-1 (PMC9479267; doi:10.1186/s12934-022-01914-1)
Supplement: Supplementary file 1 — Additional file1: Additional file results. Optimization of conditions for induction of GST fusion protein expression. Additional file methods. Molecular cloning. Production and purification of GST fusion proteins. Figure S1. Complementary DNA encoding GST-CB1414-472 fusion protein and primary amino acid sequence thereof. Figure S2. Complementary DNA (uppercase letters) encoding GST-CB1414-442 fusion protein and primary amino acid sequence thereof. Figure S3. Optimization of the conditions for the IPTG-inducible expression of fusion proteins GST, GST-CB1414-442, and GST-CB1414-472. Figure S4. Results of quantitative Western blot analysis of CB1 receptor density obtained on each of three P2 membrane samples from rat cerebral cortex tested with CB1-Af380, CB1-Af450 and CB1-ImmGs antibodies. [file 12934_2022_1914_MOESM1_ESM.pdf]

Title: Design and validation of recombinant protein standards for quantitative Western blot analysis of cannabinoid CB_1 receptor density in cell membranes: an alternative to radioligand binding methods

Miquel Saumell-Esnaola[a], Ainhoa Elejaga-Jimeno[b], Leyre Echeazarra[c,e], Leire Borrega-Román[a], Sergio Barrondo[a,f,g], Maider López de Jesús[a,f], Imanol González-Burguera[d,f], Alberto Gómez-Caballero[b], María Aranzazu Goicolea[b], Joan Sallés[a,f,g], Gontzal García del Caño[d,f,*]

Affiliations:

[a] Department of Pharmacology, Faculty of Pharmacy, University of the Basque Country UPV/EHU, Vitoria-Gasteiz, Spain.

[b] Department of Analytical Chemistry, Faculty of Pharmacy, University of the Basque Country UPV/EHU, Vitoria-Gasteiz, Spain.

[c] Department of Physiology, Faculty of Pharmacy, University of the Basque Country UPV/EHU, Vitoria-Gasteiz, Spain.

[d] Department of Neurosciences, Faculty of Pharmacy, University of the Basque Country UPV/EHU, Vitoria-Gasteiz, Spain.

[e] Bioaraba, Dispositivos Móviles para el Control de Enfermedades Crónicas, 01008 Vitoria-Gasteiz, Spain.

[f] Bioaraba, Neurofarmacología Celular y Molecular, 01008 Vitoria-Gasteiz, Spain.

[g] Centro de Investigación Biomédica en Red de Salud Mental (CIBERSAM), 28029 Madrid, Spain.

* Corresponding author. Department of Neurosciences, Faculty of Pharmacy, University of the Basque Country UPV/EHU, Paseo de la Universidad, 7, 01006 Vitoria-Gasteiz, Spain. E-mail address: gontzal.garcia@ehu.eus (G. García del Caño)

Supplementary results

Optimization of conditions for induction of GST fusion protein expression

The overexpression of recombinant proteins in bacteria can be compromised by different factors, such as nutrient depletion after long induction times or misfolding, which leads to incomplete synthesis, proteolytic degradation and/or formation of inclusion bodies [1]. Also, recombinant proteins can be toxic to the host microorganism, leading to loss of the insert-containing plasmid as a kind of selective pressure when a certain level of basal expression of recombinant protein occurs in the absence of the inducer (triggered by lactose from the culture medium due to insufficient repressor activity on the Ptac promoter). Shortcomings derived from nutrient depletion and misfolding are usually overcome by reducing temperature and/or induction time, whereas those arising from basal protein expression in the absence of the inducer can be solved by adding glucose to the preculture and during induction [2]. Taking into account these possible drawbacks, the induction was carried out in variable conditions of temperature, time and in the presence or not of glucose in the medium, in order to establish the optimal conditions for the expression of GST-CB1$_{414\text{-}442}$ and GST-CB1$_{414\text{-}472}$. Subsequently, protein products were analyzed from bacterial lysates by SDS-PAGE and Coomassie blue staining. As shown in figure S3A, induction at 25°C for 18 hours or at 37°C for 3 hours (both in the absence of glucose) were the most favorable conditions for expression of the GST and GST-CB1$_{414\text{-}442}$ constructs, although induction at 37°C for 3 hours produced considerably higher expression levels of GST-CB1$_{414\text{-}442}$ construct than induction at 25°C for 18 hours. Strikingly, the optimal conditions for the synthesis of the GST-CB1$_{414\text{-}472}$ construct varied with respect to GST and GST-CB1$_{414\text{-}442}$. Thus, only induction at 37 °C for 3 hours resulted in a single band at the expected theoretical molecular mass for GST-CB1$_{414\text{-}472}$, whereas induction at 25 °C for 18 hours yielded apparently no construct and induction at 18 °C for 18 hours produced an extra band migrating slightly above of the mass of "empty" GST protein. Of note, addition of glucose to the induction medium inhibited growth of all pGEX-6P1 clones, as reflected by the drastic decrease in the intensity of Coomassie staining, despite doubling the cell lysate load for cultures induced in presence of 2% glucose (Fig. S3A). In view that induction at 37 °C for 3 hours was the best condition for the synthesis of GST-CB1$_{414\text{-}442}$ and GST-CB1$_{414\text{-}472}$ constructs, we tested the possibility that shortening the induction time at 37 °C or increasing the temperature to 44 °C could improve the expression yield of the CB1$_{414\text{-}442}$ and GST-CB1$_{414\text{-}472}$ constructs. None of the conditions tested enhanced the expression of GST fusion

proteins compared to induction at 37 °C for 3 hours (Fig. S3B), which was chosen as the standard condition.

Supplementary methods

Molecular cloning

A pCDNA3.0 with an insert corresponding to the sequence of the human CB_1 receptor (NCBI Reference Sequence, NM_007726.5) was used as template for PCR amplification of DNA coding for $CB1_{414\text{-}442}$ and $CB1_{414\text{-}472}$ polypeptides. Briefly, 2 µL template (20 ng/µL) were brought to a final volume of 50 µL reaction mixture containing 2 mM Mg^{2+}, 0.3 mM dNTPs, 0.3 µM primer mix and 0.5 units KAPA HiFi Taq polymerase (Cat. KK2101; KAPA Biosystems, Inc., Woburn, MA, USA). PCR reactions were performed using a Bio-Rad thermocycler (Bio-Rad, MyCycler™ thermocycler system). Following a hot start for 2 min at 95 °C, 5 cycles of amplification were performed by melting at 96 °C for 30 min, annealing at 59 °C for 30 s and extending at 72 °C for 1 min, followed by 25 identical cycles except that the annealing temperature was raised to 70 °C. After a final extension step at 72 °C for 5 min, the PCR products were checked for purity and length in minigels, followed by migration in preparative agarose gels and purification using Macherey-Nagel Nucleospin® Gel and PCR Clean-Up columns (Cat. 740609; Clontech, Madrid, Spain) according to the manufacturer's instructions. To insert the purified PCR products into the pCR™-Blunt II-TOPO™ cloning plasmid, one Shot® TOP10 chemically competent bacteria (Cat. C404010; Thermo Fisher Scientific) were heat shock transformed with the PCR product-containing plasmids and cultured in on LB agar (Lennox) (Cat. 1083; Condalab, Madrid, Spain) supplemented with 50 µg/mL kanamycin (Cat. SR0092; Thermo Fisher Scientific). Individual colonies were picked and grown in kanamycin supplemented LB Broth (Lennox) (Cat. 1231; Condalab) at 37 °C for 16-18 h. Thereafter, plasmid DNA was purified using Macherey-Nagel Nucleospin® Plasmid columns (Cat. 740588; Clontech) and subjected to digest screening with BamHI (Cat. R0136S; New England Biolabs, Barcelona, Spain) and BsrGI (Cat. R0575S; New England Biolabs) enzymes. Positive clones were selected for sequencing at the STABVIDA sequencing facility (Lisbon, Portugal). For digestion-ligation cloning into the bacterial expression vector pGEX-6P1, insert-containing TOPO plasmids and the pGEX-6P1 vector were digested with BamHI and NotI (Cat. R0189L; New England Biolabs) enzymes (10 µg DNA/reaction) and the resulting products were resolved in a 1% preparative agarose gel. The fragments of TOPO vectors corresponding to the inserts carrying the coding sequences of the polypeptides $CB1_{414\text{-}442}$ and $CB1_{414\text{-}472}$ (93 and 183

bp, respectively) and the linearized pGEX-6P1 vector (4955 bp) were purified on Nucleospin Gel and PCR Clean-Up columns. Inserts were then ligated into the digested pGEX-6P1 plasmid with T4 DNA ligase (Cat. 2011A; Takara Bio Inc., Shiga, Japan) according to the manufacturer's recommendations, for 18 hours at 16 °C with a vector to insert molar ratios of 1:10. TOP10 chemically competent bacteria were transformed with the ligation products and cultured in LB-agar supplemented with 100 µg/mL of ampicillin (Cat. A9518; Sigma-Aldrich). Subsequently, we proceeded as described for the pCR™-Blunt II-TOPO™ plasmid for colony screening, except that LB Broth medium contained ampicillin (100 µg/mL) instead of kanamycin. The positive clones were sent for sequencing to verify the position of the inserts in the correct reading frame and the absence of mutations that alter the expected amino acid coding. The pGEX-P1 plasmids carrying the coding sequences for GST-$CB1_{414-442}$ and GST-$CB1_{414-472}$ proteins and the empty pGEX-P1 plasmid were transferred by heat shock to the BL21-derived bacterial strain Rosetta™(DE3)pLysS (Cat. 70956; Sigma-Aldrich). Then, the transformed bacteria were plated on LB-Agar under ampicillin (100 µg/mL) and chloramphenicol (34 µg/mL; Cat. C0378; Sigma-Aldrich) pressure to retain the transferred pGEX-6P1 and pRARE plasmids (contained in Rosetta strain for efficient translation of eukaryotic codons rarely present in bacteria), respectively. After growing overnight at 4 ºC, one clone per construction was picked and grown overnight at 37 °C in 5 mL LB Broth containing ampicillin (100 µg/mL) and chloramphenicol (34 µg/mL). A sample from this pre-culture was stored as glycerol stock at -80 °C and the rest was used for GST fusion protein production.

Production and purification of GST fusion proteins

IPTG-induced bacterial pellets were re-suspended in 6 mL Tris-Buffered Saline (TBS, 50 mM Tris-HCl, 150 mM NaCl, pH 7.6) containing bacterial protease inhibitor cocktail (54 mg/g wet weight of bacterial pellet) (Cat. P8465; Sigma-Aldrich) and 1 mM 4-(2-Aminoethyl)benzenesulfonyl fluoride hydrochloride (Cat. 76307 Pefabloc® SC; Sigma-Aldrich). Bacteria were then subjected to three sonication cycles on ice using a VibraCell® VC50T ultrasonic processor (Sonics & Materials Inc., Danburry, USA) at 50% amplitude with a 3 mm probe and 5 min rest between cycles. Each cycle consisted of 3 bursts (20 s each with 5 s on/off pulses) and 1 min rest between bursts. After sonication, Triton X-100 was added to the lysate to a final 1% (v/v) concentration followed by incubation for 30 min at 4 °C on a swing shaker and centrifugation for 30 min at 30,000 x g at 4 °C in a high-speed centrifuge (Centrikon T-42K, Kontron). GST fusion proteins were purified from the

supernatants using Pierce™ Glutathione Magnetic Agarose Beads (Cat. 78602; Thermo Fisher Scientific) following the procedure recommended by the supplier. Finally, GST fusion proteins were eluted twice to a final volume of 4 mL with 10 mM reduced glutathione (Cat. G4251; Sigma-Aldrich) in 50 mM Tris-HCl, pH 8.0, containing 1 mM DTT.

References

1. Bowden GA, Georgiou G. Folding and aggregation of β-lactamase in the periplasmic space of Escherichia coli. J Biol Chem. United States; 1990;265:16760–6.
2. Miroux B, Walker JE. Over-production of proteins in Escherichia coli: Mutant hosts that allow synthesis of some membrane proteins and globular proteins at high levels. J Mol Biol [Internet]. 1996;260:289–98. Retrieved from: https://www.sciencedirect.com/science/article/pii/S002228369690399X

```
cggctcgtataatgtgtggaattgtgagcggataacaatttcacacaggaaacagtattc     -1

ATGTCCCCTATACTAGGTTATTGGAAAATTAAGGGCCTTGTGCAACCCACTCGACTTCTT     60
MetSerProIleLeuGlyTyrTrpLysIleLysGlyLeuValGlnProThrArgLeuLeu     20

TTGGAATATCTTGAAGAAAAATATGAAGAGCATTTGTATGAGCGCGATGAAGGTGATAAA    120
LeuGluTyrLeuGluGluLysTyrGluGluHisLeuTyrGluArgAspGluGlyAspLys     40

TGGCGAAACAAAAAGTTTGAATTGGGTTTGGAGTTTCCCAATCTTCCTTATTATATTGAT    180
TrpArgAsnLysLysPheGluLeuGlyLeuGluPheProAsnLeuProTyrTyrIleAsp     60

GGTGATGTTAAATTAACACAGTCTATGGCCATCATACGTTATATAGCTGACAAGCACAAC    240
GlyAspValLysLeuThrGlnSerMetAlaIleIleArgTyrIleAlaAspLysHisAsn     80

ATGTTGGGTGGTTGTCCAAAAGAGCGTGCAGAGATTTCAATGCTTGAAGGAGCGGTTTTG    300
MetLeuGlyGlyCysProLysGluArgAlaGluIleSerMetLeuGluGlyAlaValLeu    100

GATATTAGATACGGTGTTTCGAGAATTGCATATAGTAAAGACTTTGAAACTCTCAAAGTT    360
AspIleArgTyrGlyValSerArgIleAlaTyrSerLysAspPheGluThrLeuLysVal    120

GATTTTCTTAGCAAGCTACCTGAAATGCTGAAAATGTTCGAAGATCGTTTATGTCATAAA    420
AspPheLeuSerLysLeuProGluMetLeuLysMetPheGluAspArgLeuCysHisLys    140

ACATATTTAAATGGTGATCATGTAACCCATCCTGACTTCATGTTGTATGACGCTCTTGAT    480
ThrTyrLeuAsnGlyAspHisValThrHisProAspPheMetLeuTyrAspAlaLeuAsp    160

GTTGTTTTATACATGGACCCAATGTGCCTGGATGCGTTCCCAAAATTAGTTTGTTTTAAA    540
ValValLeuTyrMetAspProMetCysLeuAspAlaPheProLysLeuValCysPheLys    180

AAACGTATTGAAGCTATCCCACAAATTGATAAGTACTTGAAATCCAGCAAGTATATAGCA    600
LysArgIleGluAlaIleProGlnIleAspLysTyrLeuLysSerSerLysTyrIleAla    200

TGGCCTTTGCAGGGCTGGCAAGCCACGTTTGGTGGTGGCGACCATCCTCCAAAATCGGAT    660
TrpProLeuGlnGlyTrpGlnAlaThrPheGlyGlyGlyAspHisProProLysSerAsp    220

                              BamHI   Forward primer
CTGGAAGTTCTGTTCCAGGGGCCCCTGGGATCCTGTGAAGGCACTGCGCAGCCTCTGGAT    720
LeuGluValLeuPheGlnGlyProLeuGlySerCysGluGlyThrAlaGlnProLeuAsp    240
             PreScission cleavage

AACAGCATGGGGGACTCGGACTGCCTGCACAAACACGCAAACAATGCAGCCAGTGTTCAC    780
AsnSerMetGlyAspSerAspCysLeuHisLysHisAlaAsnAsnAlaAlaSerValHis    260

AGGGCCGCAGAAAGCTGCATCAAGAGCACGGTCAAGATTGCCAAGGTAACCATGTCTGTG    840
ArgAlaAlaGluSerCysIleLysSerThrValLysIleAlaLysValThrMetSerVal    280

          Reverse primer        NotI
TCCACAGACACGTCTGCCGAGGCTCTGTGAgcggccgcatcgtgactgactgacgatctg    900
SerThrAspThrSerAlaGluAlaLeu *                                   289

cctcgcgcgtttcggtgatgacggtgaaaa                                  930
```

Fig S1 Complementary DNA (uppercase letters) encoding GST-$CB1_{414\text{-}472}$ fusion protein and primary amino acid sequence thereof. Shaded nucleotides correspond to the hybridization sites for the Fw and Rv primers used to obtain the inserts flanked by BamHI and NotI restriction sites. The portions of the amino acid sequence underlined by solid and dashed lines correspond to the sequences of the GST tag and the $CB1_{414\text{-}472}$ fragment, respectively. The residue corresponding to serine 414 in the human CB_1 receptor sequence is shown in bold. The shaded amino acid sequence (LeuPheGlnGlyPro) corresponds to the consensus cleavage site for the PreScission protease.

```
cggctcgtataatgtgtggaattgtgagcggataacaatttcacacaggaaacagtattc     -1

ATGTCCCCTATACTAGGTTATTGGAAAATTAAGGGCCTTGTGCAACCCACTCGACTTCTT     60
MetSerProIleLeuGlyTyrTrpLysIleLysGlyLeuValGlnProThrArgLeuLeu     20

TTGGAATATCTTGAAGAAAAATATGAAGAGCATTTGTATGAGCGCGATGAAGGTGATAAA    120
LeuGluTyrLeuGluGluLysTyrGluGluHisLeuTyrGluArgAspGluGlyAspLys     40

TGGCGAAACAAAAAGTTTGAATTGGGTTTGGAGTTTCCCAATCTTCCTTATTATATTGAT    180
TrpArgAsnLysLysPheGluLeuGlyLeuGluPheProAsnLeuProTyrTyrIleAsp     60

GGTGATGTTAAATTAACACAGTCTATGGCCATCATACGTTATATAGCTGACAAGCACAAC    240
GlyAspValLysLeuThrGlnSerMetAlaIleIleArgTyrIleAlaAspLysHisAsn     80

ATGTTGGGTGGTTGTCCAAAAGAGCGTGCAGAGATTTCAATGCTTGAAGGAGCGGTTTTG    300
MetLeuGlyGlyCysProLysGluArgAlaGluIleSerMetLeuGluGlyAlaValLeu    100

GATATTAGATACGGTGTTTCGAGAATTGCATATAGTAAAGACTTTGAAACTCTCAAAGTT    360
AspIleArgTyrGlyValSerArgIleAlaTyrSerLysAspPheGluThrLeuLysVal    120

GATTTTCTTAGCAAGCTACCTGAAATGCTGAAAATGTTCGAAGATCGTTTATGTCATAAA    420
AspPheLeuSerLysLeuProGluMetLeuLysMetPheGluAspArgLeuCysHisLys    140

ACATATTTAAATGGTGATCATGTAACCCATCCTGACTTCATGTTGTATGACGCTCTTGAT    480
ThrTyrLeuAsnGlyAspHisValThrHisProAspPheMetLeuTyrAspAlaLeuAsp    160

GTTGTTTTATACATGGACCCAATGTGCCTGGATGCGTTCCCAAAATTAGTTTGTTTTAAA    540
ValValLeuTyrMetAspProMetCysLeuAspAlaPheProLysLeuValCysPheLys    180

AAACGTATTGAAGCTATCCCACAAATTGATAAGTACTTGAAATCCAGCAAGTATATAGCA    600
LysArgIleGluAlaIleProGlnIleAspLysTyrLeuLysSerSerLysTyrIleAla    200

TGGCCTTTGCAGGGCTGGCAAGCCACGTTTGGTGGTGGCGACCATCCTCCAAAATCGGAT    660
TrpProLeuGlnGlyTrpGlnAlaThrPheGlyGlyGlyAspHisProProLysSerAsp    220
                                      BamHI
                                       ▼        Forward primer
CTGGAAGTTCTGTTCCAGGGGCCCCTGGGATCCTGTGAAGGCACTGCGCAGCCTCTGGAT    720
LeuGluValLeuPheGlnGlyProLeuGlySerCysGluGlyThrAlaGlnProLeuAsp    240
                    ▲
         PreScission cleavage
                                                  Reverse primer
AACAGCATGGGGGACTCGGACTGCCTGCACAAACACGCAAACAATGCAGCCAGTGTTTGA    780
AsnSerMetGlyAspSerAspCysLeuHisLysHisAlaAsnAsnAlaAlaSerVal *    259
NotI
  ▼
gcggccgcatcgtgactgactgacgatctgcctcgcgcgtttcggtgatgacggtgaaaa    840
```

Fig S2 Complementary DNA (uppercase letters) encoding GST-$CB1_{414\text{-}442}$ fusion protein and primary amino acid sequence thereof. Shaded nucleotides correspond to the hybridization sites for the Fw and Rv primers used to obtain the inserts flanked by BamHI and NotI restriction sites. The portions of the amino acid sequence underlined by solid and dashed lines correspond to the sequences of the GST tag and the $CB1_{414\text{-}442}$ fragment, respectively. The residue corresponding to serine 414 in the human CB_1 receptor sequence is shown in bold. The shaded amino acid sequence (LeuPheGlnGlyPro) corresponds to the consensus cleavage site for the PreScission protease.

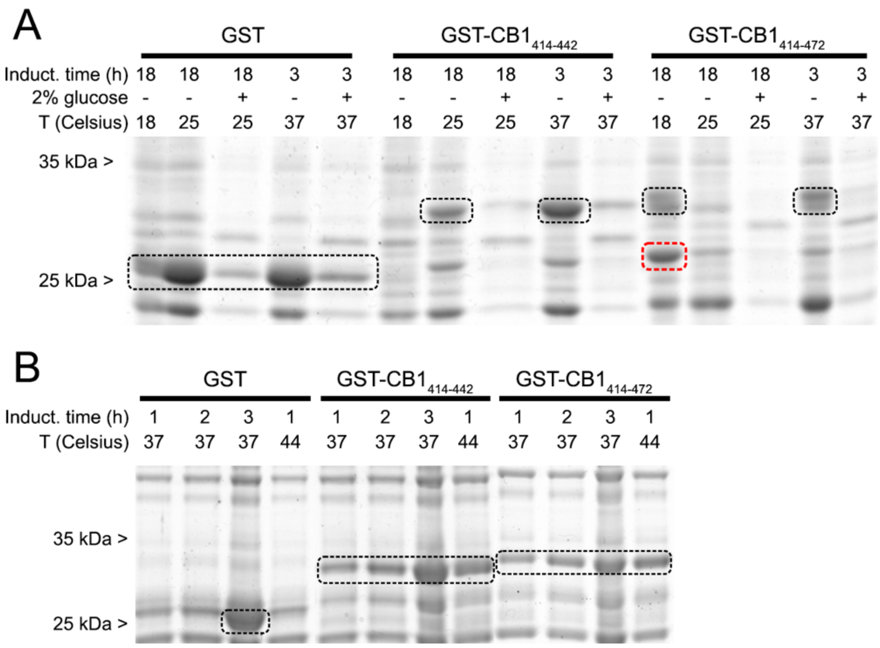

Fig S3 Optimization of the conditions for the IPTG-inducible expression of fusion proteins GST, GST-CB1$_{414\text{-}442}$, and GST-CB1$_{414\text{-}472}$ in Rosetta^{TM} (DE3) pLysS bacteria. **A** SDS-PAGE and Coomassie blue staining of whole-cell pellet lysates from bacterial cultures subjected to variable conditions of temperature and induction time and grown in the absence or presence of 2% glucose in the culture medium. **B** Same analysis as in A to test the impact of induction time and high temperature. Black dashed lines delineate protein bands corresponding to GST fusion proteins that migrate according to their theoretical molecular mass. The band outlined by the red dashed line corresponds to a protein resulting from the incomplete synthesis of the GST-CB1$_{414\text{-}442}$ construct or a product of its proteolytic degradation.

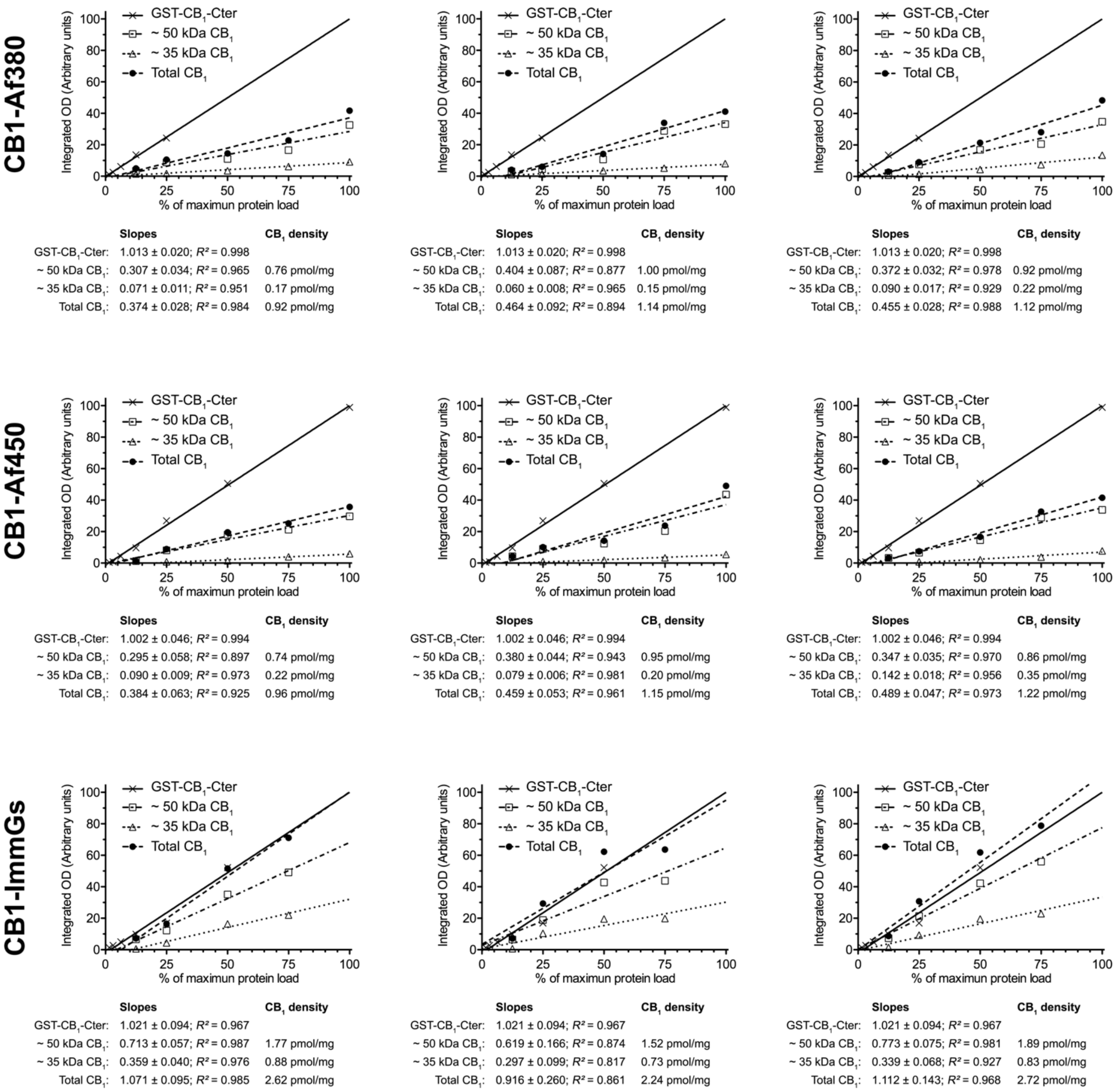

Figure S4. Results of quantitative Western blot analysis of CB_1 receptor density obtained on each of three crude synaptosome samples (P2 membranes) from rat cerebral cortex tested with CB1-Af380, CB1-Af450 and CB1-ImmGs antibodies. Graphs show linear regression analysis of the integrated optical density (Integrated OD) values of immunoreactivity corresponding to the endogenous CB_1 receptor in P2 membranes and to the recombinant construct $GST\text{-}CB1_{414\text{-}472}$. The integrated OD values were obtained by densitometric analysis of the immunoreactive bands produced by the endogenous CB_1 receptor at ~50 and ~35 kDa and by the recombinant standard $GST\text{-}CB1_{414\text{-}472}$ (subtracting the value of the non-specific signal produced by the $GST\text{-}CB1_{414\text{-}442}$ protein) at increasing sample loads. The slopes obtained for each of the three P2 samples run side by side, together with the $GST\text{-}CB1_{414\text{-}472}$ and $GST\text{-}CB1_{414\text{-}442}$ recombinant proteins, (see Fig. 5) are shown. The molar amounts of the ~50 and ~35 kDa CB_1 receptor species and of the total CB_1 receptor per unit mass of P2 protein estimated from slope values are shown below each graph.

FULL IMAGES OF GELS AND WESTERN BLOTS

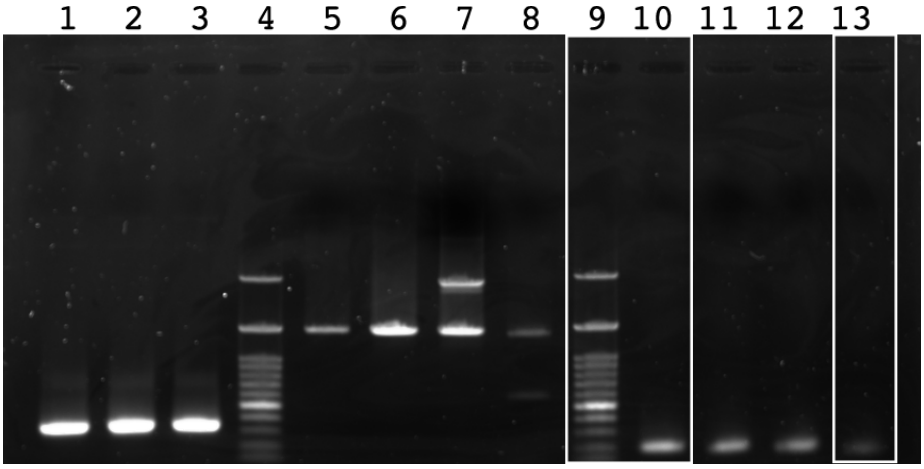

Full agarose gel electrophoresis image of the PCR products shown in Figure 2A constructed with the three boxed lanes corresponding to the DNA ladder (lane 9) and the PCR amplicons encoding the $CB1_{414-472}$ (lane 10) and $CB1_{414-442}$ (lane 13) fragments of the cytosolic tail of the human CB_1 receptor. The rest of the signals correspond to PCR products unrelated to the present study that were tested on the same agarose gel.

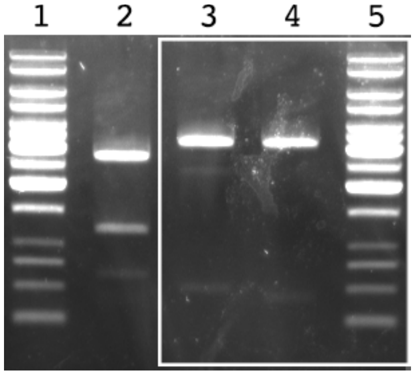

Full agarose gel electrophoresis image of BamHI/BsrGI restriction analysis shown in Figure 2B constructed with the three boxed lanes corresponding to pCR™-Blunt II-TOPO™ vector clones with restriction maps consistent with DNA inserts coding for $CB1_{414-472}$ and $CB1_{414-442}$ (lanes 3 and 4 respectively) and DNA ladder (lane 5).

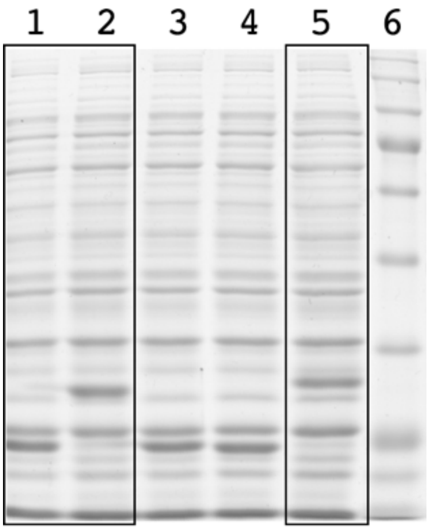

Full image of SDS-PAGE and Coomassie blue staining of whole-cell pellet lysates shown in Figure 3B constructed with the three boxed lanes corresponding to clones overexpressin GST (lane 1), GST-CB1$_{414-442}$ (lane 2) and GST-CB1$_{414-472}$ (lane 3) proteins. Lanes 3 and 4 are lysates from clones unrelated to the present study that were tested on the same gel but removed from Figure 2B. Lane 6 corresponds to the DNA ladder, which was also removed.

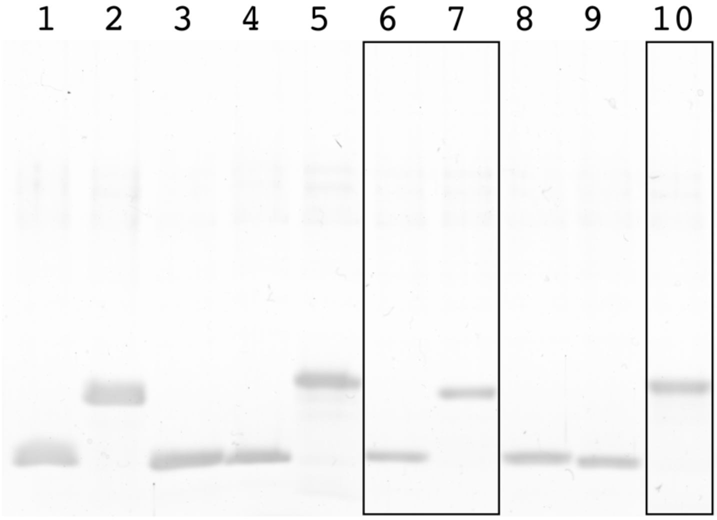

Full image of SDS-PAGE and Coomassie blue staining shown in Figure 3C. The boxed lanes 6, 7 and 10 corresponding to affinity purified GST, GST-CB1$_{414-442}$ and GST-CB1$_{414-472}$ proteins were used for the illustration. Removed lanes correspond either to higher loads of the same constructs (lanes 1, 2 and 5) or to affinity purified recombinant proteins unrelated to the present study (3-4 and 8-9).

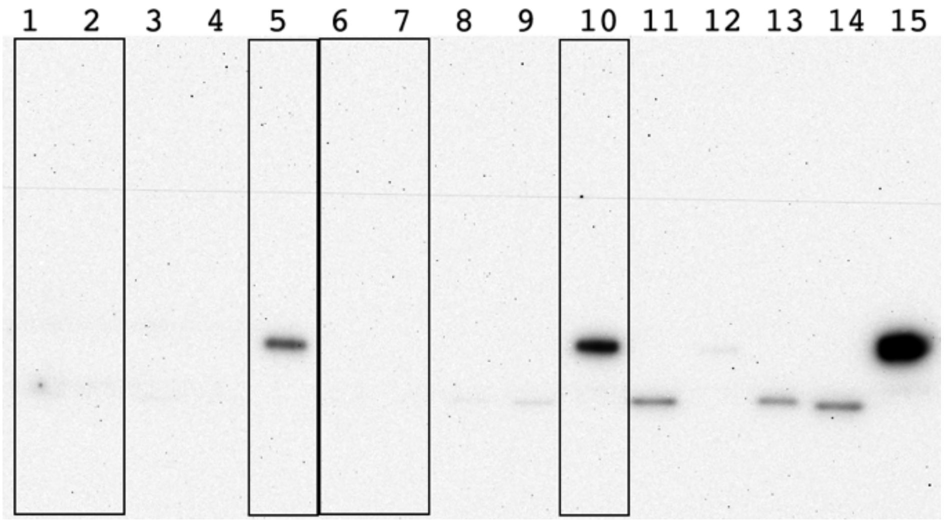

Full image of the immunoblot shown in figure 3D. The boxed lanes 1, 2, 5, 6, 7 and 10 corresponding to affinity purified GST (lanes 1 and 6) , GST-CB1$_{414-442}$ (lanes 2 and 7) and GST-CB1$_{414-472}$ (lanes 5 and 10) proteins were used for the illustration. Removed lanes correspond either to higher loads of the same constructs (lanes 11, 12 and 15) or to affinity purified recombinant proteins unrelated to the present study (3-4, 8-9 and 13-14).

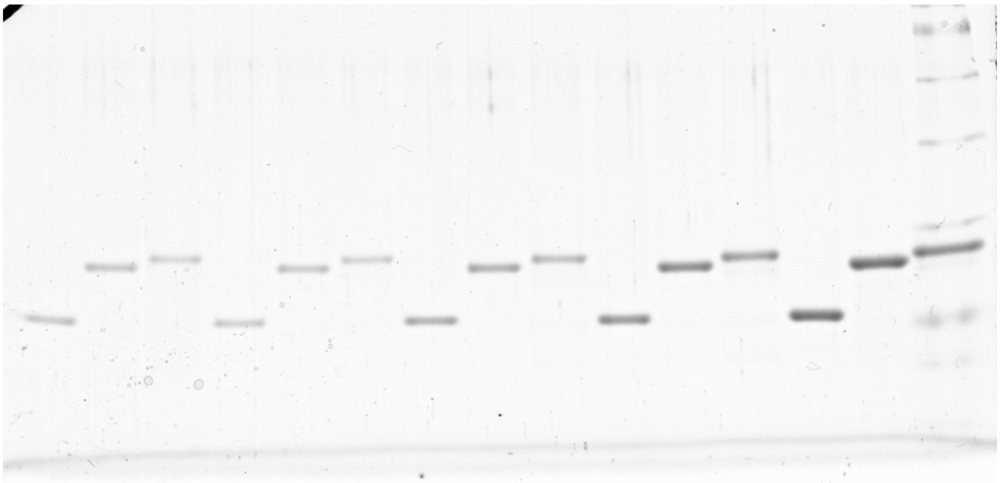

Full image of SDS-PAGE and Coomassie blue staining shown in Figure 4.

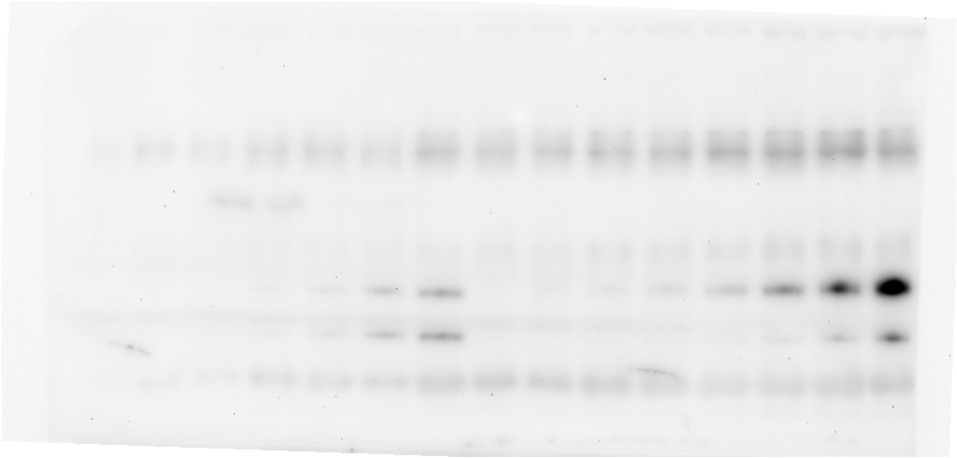

Full image of the immunoblot shown in figure 5A.

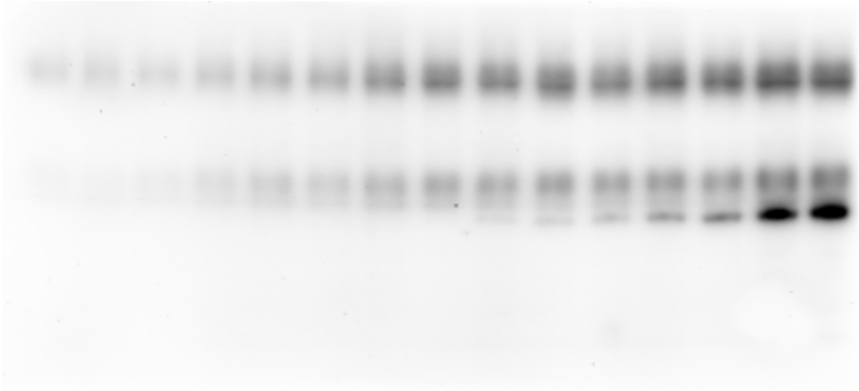

Full image of the immunoblot shown in figure 5D.

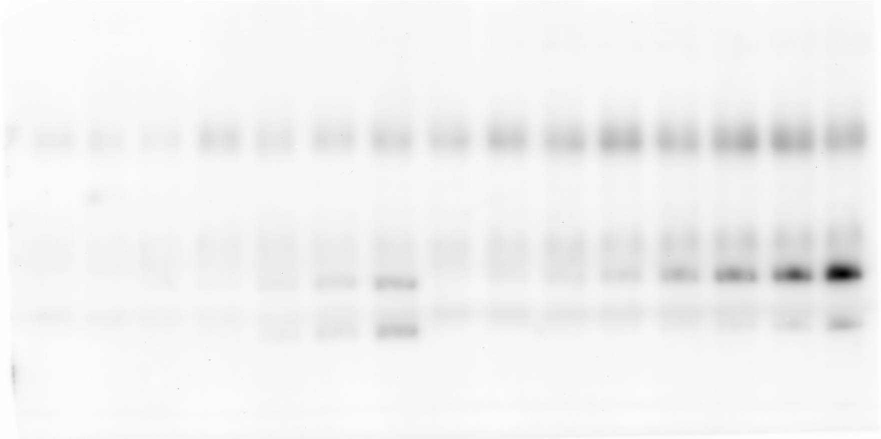

Full image of the immunoblot shown in figure 5G.
