# Supplementary figures and images for: Design and validation of recombinant protein standards for quantitative Western blot analysis of cannabinoid CB1 receptor density in cell membranes: an alternative to radioligand binding methods
Source: Microb Cell Fact. 2022 Sep 15;21:192. doi: 10.1186/s12934-022-01914-1 (PMC9479267; doi:10.1186/s12934-022-01914-1)

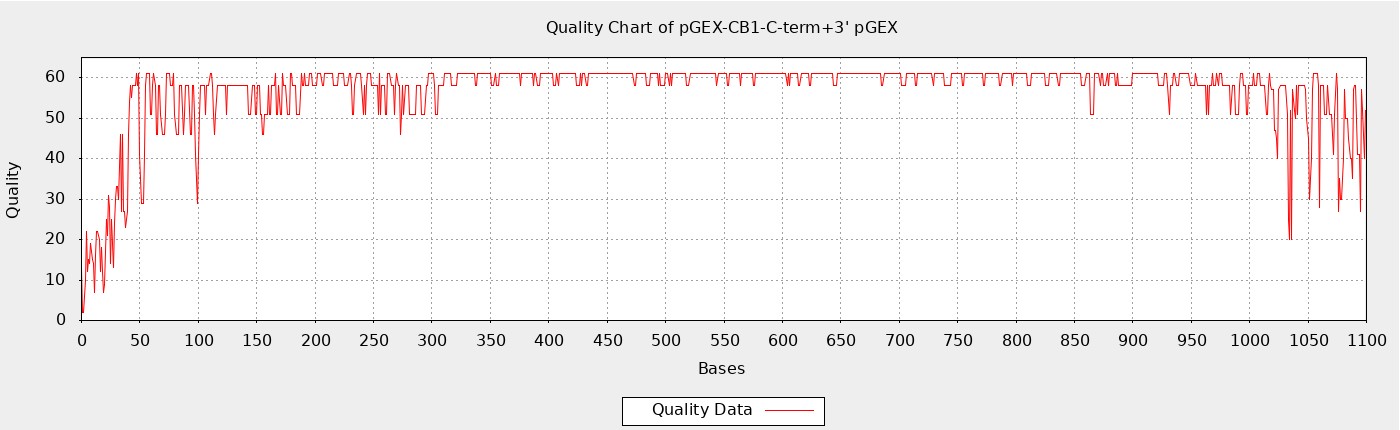

Supplement: Supplementary file 2 — Additional file2: zip-compressed files of [i] theoretical sequences corresponding to pGEX-6P1 plasmids with DNA constructs inserted downstream the GST gene for the inducible expression of GST fusion proteins GST-CB1414-472 and GST-CB1414-442 and [ii] results of sequencing [file 12934_2022_1914_MOESM2_ESM.zip › 02 Sequencing/3'pGEX pGEX_hCB1_414_472.jpeg]

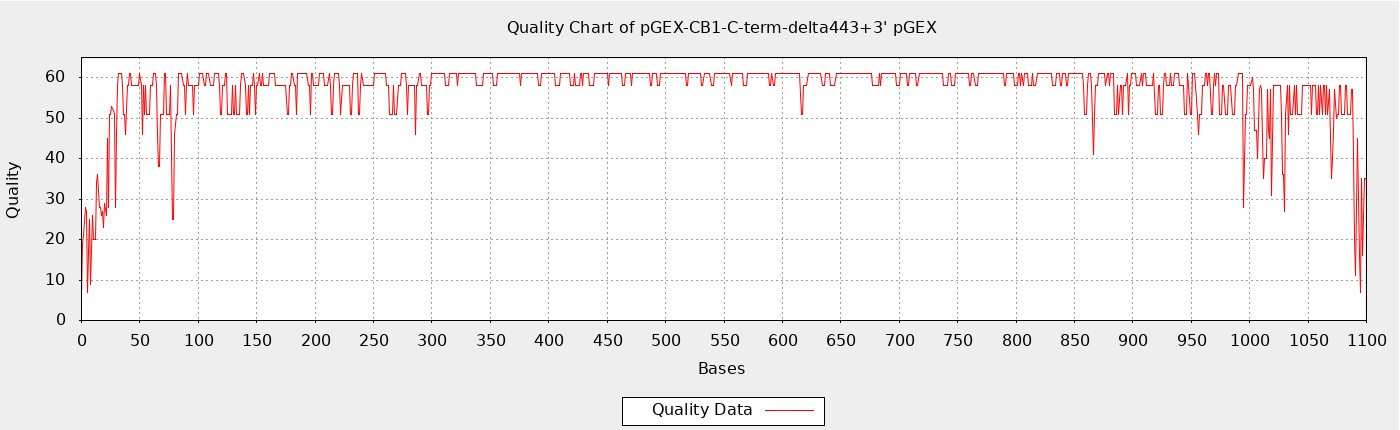

Supplement: Supplementary file 2 — Additional file2: zip-compressed files of [i] theoretical sequences corresponding to pGEX-6P1 plasmids with DNA constructs inserted downstream the GST gene for the inducible expression of GST fusion proteins GST-CB1414-472 and GST-CB1414-442 and [ii] results of sequencing [file 12934_2022_1914_MOESM2_ESM.zip › 02 Sequencing/3'pGEX pGEX_hCB1_414_442.jpeg]
